# Application of UPLC-MS/MS-Based Widely Targeted Metabolomics Reveals Metabolic Reprogramming During Post-Harvest Storage of *Ehretia macrophylla* Fruits

**DOI:** 10.3390/foods15173154

**Published:** 2026-09-05

**Authors:** Xining Geng, Mengyao Luo, Fengqin Huang, Shuaizheng Qi, Lihua Xie, Meiyu Li, Minghui Chen, Congping Xu, Shiping Cheng

**Affiliations:** 1 Henan Key Laboratory of Germplasm Innovation and Utilization of Eco-Economic Woody Plant, Pingdingshan University, Pingdingshan 467000, China; geng_xn@yeah.net (X.G.); hfq4117005070@stu.xjtu.edu.cn (F.H.); 13051857171@163.com (S.Q.); xielihua0227@163.com (L.X.); meiyulily@163.com (M.L.); cmh_abc@126.com (M.C.); 2 School of Life Science and Technology, Wuhan Polytechnic University, Wuhan 430023, China; lmy15516252313@126.com

**Keywords:** *Ehretia macrophylla*, UPLC-MS/MS, widely targeted metabolomics, post-harvest storage, metabolic reprogramming, flavonoid metabolism

## Abstract

*Ehretia macrophylla* fruit is a traditionally used but underutilized functional resource that is noted for its diverse phytochemical composition, which includes flavonoids and polyphenols. However, the systematic metabolic transformations that occur in the fruit during post-harvest storage—particularly those associated with its traditional processing into dark fruit tea—remain largely unknown, limiting efforts to optimize quality and processing strategies. In the study, to address this gap, we employed a widely targeted metabolomics approach based on ultra-performance liquid chromatography–tandem mass spectrometry (UPLC-MS/MS) to dynamically profile the fruit metabolome across four storage stages (T0, T4, T8, T12). A total of 1101 metabolites were putatively annotated, and day 8 (T8) emerged as a potential pronounced metabolic inflection point. From T8 onward, 30–40% of metabolites showed differential accumulation, characterized by a gradual rise in nutrition-relevant lipids, amino acids, and vitamins. In contrast, key antioxidant-related metabolites, including phenolics and flavonoids, increased transiently before T8 but declined sharply thereafter. These coordinated shifts correlated with an increased representation of lipid- and alkaloid-related metabolic pathways, whereas flavonoid-associated features showed a relative decline. Collectively, these findings provide insights into the biochemical processes potentially associated with fruit blackening and the marked decline in antioxidant capacity during traditional processing. Our study provides the first metabolic blueprint connecting traditional processing practices with phased metabolic remodeling, offering a scientific foundation for quality assessment and the development of informed post-harvest strategies for processing *E. macrophylla* fruit.

## 1. Introduction

The increasing global demand for natural products and functional foods has motivated an interest in underutilized plant species that possess both nutritional and medicinal value [1]. In this context, fruits rich in phytochemicals—particularly flavonoids and polyphenols—are recognized not only as dietary components but also as promising sources of compounds associated with mitigating oxidative stress and chronic inflammation [2,3]. Consequently, systematic metabolomic investigations of such species, especially during critical post-harvest stages, are essential for unlocking their full potential in the food, pharmaceutical, and nutraceutical industries [4].

*Ehretia macrophylla*, a deciduous tree of the Boraginaceae family, is a typical yet underexploited resource with considerable development prospects [5]. Its dried fruits have been traditionally used to alleviate symptoms of coughing and sphagitis [6] and modern phytochemical analyses have corroborated these traditional uses, revealing exceptionally high contents of flavonoids and phenolic compounds such as gallic and ellagic acids [7]. The fruit is rich in flavonoids and phenolic acids, which have been associated with antioxidant, anti-proliferative, anti-inflammatory, hypoglycemic, and lung-protective activities in previous in vitro studies [8,9]. Additionally, its fruit polysaccharides may act as a potential prebiotic for regulating colonic health [6]. The convergence of its historical use as both a food and medicine with contemporary scientific validation underscores *E. macrophylla* fruit as a prime candidate for systematic development and further investigation.

Metabolomics has become an indispensable tool for comprehensively characterizing the dynamic biochemical landscape of biological systems [10], playing a pivotal role in post-harvest research for elucidating metabolic shifts that determine fruit quality, shelf-life, and the stability of bioactive compounds. Integrated approaches—particularly widely targeted metabolomics coupled with UPLC-MS/MS—enable the high-throughput and precise quantification of hundreds of metabolites, offering unprecedented insights into metabolic reprogramming during processing, storage, and drying [11,12]. Such studies have been successfully conducted for various fruits, including *Rosa roxburghii* and avocado, clarifying the pathways underlying nutrient accumulation and degradation [13,14]. Recent advances have further extended metabolomic applications to dissect the interplay between primary and secondary metabolic networks during post-harvest senescence in fruits such as blueberry and lychee, revealing that sugar–acid balance and phenylpropanoid flux are tightly coupled with antioxidant capacity fluctuations [15,16]. However, a critical gap persists between the documented bioactivities of *E. macrophylla* fruit and an in-depth understanding of the evolution of its post-harvest metabolite profile. Current research has primarily focused on the identification and activity assessment of extracts from fresh or minimally processed materials, alongside cultivation optimization [8,9,17,18]. Notably, the transformative biochemical processes occurring during traditional preparation methods—such as sun-drying into dark fruit tea, which is integral to its folk medicinal applications—have been systematically overlooked. The conspicuous color change from yellow in fresh fruit to black in dried products suggests extensive biochemical transformations, likely involving the oxidation, polymerization, and degradation of key metabolites. Nevertheless, the specific metabolic pathways potentially involved in this color transition, as well as the concomitant changes in bioactive constituents and their associated antioxidant functions, remain largely unexplored. More critically, it is unclear whether the decline in antioxidant activity during storage is primarily attributable to the degradation of non-enzymatic antioxidants (phenolics and flavonoids) or to the inactivation of antioxidant enzymes—a distinction that carries substantial implications for post-harvest processing optimization. This knowledge gap constitutes a major bottleneck, as it hinders the scientific optimization of post-harvest handling to maximally retain or even directionally enhance its functional components.

In this study, we bridge this pronounced gap by investigating the global metabolic changes in *E. macrophylla* fruits during post-harvest storage. A UPLC-MS/MS-based, widely targeted metabolomics approach was employed to systematically analyze the dynamic metabolic profiles from the near-mature stage through indoor shade-drying to complete blackening. Our work characterizes the metabolic transformations during traditional processing, thereby providing a scientific foundation for quality control, standardized processing, and value-added utilization of *E. macrophylla* fruits.

## 2. Materials and Methods

### 2.1. Materials and Reagents

Plant material (*E. macrophylla* fruits) was obtained from the research base of Pingdingshan University (Pingdingshan, Henan, China), as described in Section 2.2.

For physiological and biochemical assays, assay kits for peroxidase (POD), superoxide dismutase (SOD), and catalase (CAT) were purchased from Nanjing Jiancheng Bioengineering Institute (Nanjing, China). Kits for total sugars (NMW0206, 96T), soluble sugars (NMW0209, 96T), polysaccharides (NMW0207, 96T), ascorbic acid (NMW0401, 96T), and free fatty acids (NMW1044, 96T) were obtained from Normickoda Biotechnology Co., Ltd. (Wuhan, China). The total antioxidant capacity assay kit (BC1315, 100T/96S) was sourced from Solarbio Science & Technology Co., Ltd. (Beijing, China). Sodium hydroxide, phenolphthalein, and other routine chemicals were obtained from Sinopharm Chemical Reagent Co., Ltd. (Shanghai, China). Standards for calibration curves, including rutin, gallic acid, Trolox, and linalool, were purchased from Sigma-Aldrich (St. Louis, MO, USA). Cyanidin-3-O-glucoside standard was obtained from Yuanye Bio-Technology Co., Ltd. (Shanghai, China). Folin–Ciocalteu reagent, ABTS [2,2′-azino-bis(3-ethylbenzothiazoline-6-sulfonic acid)], DPPH (1,1-diphenyl-2-picrylhydrazyl), and vanillin were procured from Macklin Biochemical Co., Ltd. (Shanghai, China).

For metabolomics analysis, LC-MS-grade methanol, acetonitrile, and formic acid were obtained from Merck KGaA (Darmstadt, Germany). Ultrapure water was produced using a Milli-Q Advantage A10 system (Millipore, Bedford, MA, USA). For targeted validation, authentic standards of hesperetin 5-O-glucoside, apigenin 5-O-glucoside, kaempferol 3-O-glucoside, cinnamic acid, N-Feruloylputrescine, and Glu-Phe were purchased from Sigma-Aldrich, Ltd. (St. Louis, MO, USA), with purity ≥ 98% for all standards. All other chemicals and reagents were of analytical grade and used without further purification.

### 2.2. Plant Material and Sample Preparation

Mature fruits of *E. macrophylla* were harvested in July 2024 from the research base of Pingdingshan University, Pingdingshan City, Henan Province, China. Fruits of uniform size and maturity were selected from six individual trees to ensure genetic representativeness. The selected fruits were randomly allocated into three independent storage trays, with each tray serving as one biological replicate (*n* = 3). Each tray contained fruits pooled from all six trees and thoroughly mixed to minimize tree-to-tree variation, and the three trays were placed separately within the same controlled-environment room to ensure independent biological representation. The fruits were subjected to natural shade-drying by being placed in a temperature-controlled laboratory maintained at 26 ± 1 °C, with relative humidity maintained at 55 ± 5% using a dehumidifier (DJ-1501E, Deye, Hangzhou, Zhejiang, China). Fruit weight loss was monitored daily throughout the storage period using a precision balance, and the results are presented in Table S1. The initial moisture content of fresh fruits was determined by oven-drying at 105 °C to constant weight, and moisture content at each time point (T0, T4, T8, T12) was determined using the same method to monitor water loss dynamics (Table S1).

Sampling was performed at 0 (fresh control), 4, 8, and 12 (d) post-harvest, constituting four treatment groups (hereinafter referred to as T0, T4, T8, and T12). For each time point, fruits were randomly collected from three independent storage trays (each tray containing fruits from all six trees, pooled and thoroughly mixed), with each tray serving as one independent biological replicate (*n* = 3). From each replicate, 30 fruits were randomly taken and immediately pooled into a single composite sample per tray to minimize within-tray fruit-to-fruit variation. The pooled sample was then divided into aliquots for the various analyses described below (physiological measurements, primary metabolite quantification, secondary metabolite quantification, and metabolomics). All aliquots were immediately snap-frozen in liquid nitrogen, and then transferred to a −80 °C ultra-low-temperature freezer for subsequent analysis. Thus, for every analytical endpoint, each biological replicate (tray) yielded a single data point, ensuring that the statistical unit of analysis was the biological replicate (*n* = 3) rather than the individual fruit.

### 2.3. Physiological Measurements

The activities of antioxidant enzymes, including peroxidase (POD), superoxide dismutase (SOD), and catalase (CAT), were determined following the instructions of the respective assay kits provided by the Nanjing Jiancheng Bioengineering Institute (Nanjing, China).

For all physiological and biochemical assays described in Section 2.3 and Section 2.4, each biological replicate (tray) was represented by a single composite sample prepared from the 30 pooled fruits as described in Section 2.2. For each assay, measurements were performed on aliquots from the composite sample of each biological replicate. Where applicable, technical replicates (duplicate or triplicate measurements from the same aliquot) were averaged before statistical analysis, so that each biological replicate contributed a single data point to the final dataset. All assays were therefore conducted with three independent biological replicates (*n* = 3 per time point).

### 2.4. Quantitative Analysis of Primary Metabolites

*E. macrophylla* fruits are rich in primary metabolites. This study systematically quantified the contents of six key metabolite classes: total sugar (TS), soluble sugar (SS), polysaccharides (PSs), total acid (TA), ascorbic acid (AA), free fatty acids (FFAs), and total amino acids (TAAs). The activities of antioxidant enzymes, including peroxidase (POD), superoxide dismutase (SOD), and catalase (CAT), were determined following the instructions of the respective assay kits provided by the Nanjing Jiancheng Bioengineering Institute (Nanjing, China) [19].

#### 2.4.1. Determination of Total Sugar (TS) Content

TS content was determined using the DNS colorimetric method. First, total sugars in the sample were converted to reducing sugars via acid hydrolysis. Subsequently, the reducing sugars reacted with the DNS reagent under alkaline conditions to form a reddish-brown amino complex. This product has a characteristic absorption peak at 540 nm, and its absorbance exhibits a linear relationship with reducing sugar content within a specific range, allowing for calculation via a standard curve [20]. Specific procedures followed the kit instructions (NMW0206, 96T, Normickoda, Wuhan, China).

#### 2.4.2. Determination of Soluble Sugar (SS) Content

SS content was determined using the anthrone colorimetric method. Sugars were dehydrated by concentrated sulfuric acid to form furfural or hydroxymethylfurfural derivatives, which then reacted with anthrone to form a bluish-green complex. The absorbance was measured at 620 nm. This absorbance value shows a good linear relationship with sugar concentration within the set range, enabling quantitative analysis [21]. Experimental steps strictly adhered to the kit instructions (NMW0209, 96T, Normickoda, Wuhan, China).

#### 2.4.3. Determination of Polysaccharide (PS) Content

PSs were enriched from the samples using water extraction and alcohol precipitation, and the PS content in the extract was quantified using the phenol–sulfuric acid colorimetric method [22]. This operation was performed according to the kit instructions (NMW0207, 96T, Normickoda, Wuhan, China).

#### 2.4.4. Determination of Total Acid (TA) Content

TA content was determined using acid–base titration according to GB 12456-2021 [23]. Briefly, 200 g of the sample was homogenized with an equal volume of boiled and cooled distilled water [24]. Then, 50 mL of the filtrate was mixed with 60 mL of distilled water and 3–4 drops of phenolphthalein indicator. The mixture was titrated with a standardized 0.1 mol/L NaOH solution until a faint pink color persisted for 30 s. The consumed volume (*V*_1_) was recorded alongside a blank control (V_2_). Total acidity was calculated using the following formula: *X* = [*c* × (*V*_1_ − *V*_2_) × *K* × *F*/*m*] × 1000, where X is the total acidity (g/kg), c is the NaOH concentration (mol/L), m is the sample mass (g), F is the dilution factor, and K is the organic acid conversion coefficient. Wuhan Normickoda Biotechnology Co., Ltd., assisted with this part of the experiment.

#### 2.4.5. Determination of Ascorbic Acid (AA) Content

AA content was determined using a colorimetric method. AA reduces Fe^3+^ to Fe^2+^, and Fe^2+^ complexes with bathophenanthroline (BP) to form a red chelate. The absorbance was measured at 534 nm, and this absorbance is positively correlated with AA content, allowing for quantitative analysis [25]. This experimental process strictly followed the kit instructions (NMW0401, 96T, Normickoda, Wuhan, China).

#### 2.4.6. Determination of Free Fatty Acid (FFA) Content

An enzyme-coupled chromogenic assay was used to determine FFA content. Under the sequential catalysis of acyl-CoA synthetase (ACS) and acyl-CoA oxidase (ACO), FFAs were converted to H_2_O_2_, which then reacted with Amplex Red to generate resorufin. The absorbance at the characteristic wavelength was measured, and this absorbance value is proportional to FFA content, enabling quantification [26]. The specific steps of this process followed the kit instructions (NMW1044, 96T, Normickoda, Wuhan, China).

#### 2.4.7. Determination of Total Amino Acid (TAA) Content

TAA content was determined via a copper ion complex colorimetric method. Briefly, amino acids reacted with Cu^2+^ under alkaline conditions to generate stable blue-green complexes. The absorbance was measured at 650 nm, and the TAA concentration was quantified using a glycine standard curve within the linear range [27]. All experimental operations were conducted strictly following the manufacturer’s instructions for the commercial kit (A026-1-1, 80 assays/78 samples, Nanjing Jiancheng Bioengineering Institute, Nanjing, China).

#### 2.4.8. Determination of Antioxidant Activity

Total antioxidant capacity was measured according to the instructions for the assay kit (BC1315, 100T/96S, Solarbio, Beijing, China) [28]. The sample was co-incubated with reaction buffer and chromogenic agent at 37 °C for 10 min, and the absorbance was measured at 593 nm. A standard curve was plotted using the standard, and the results are expressed as the Trolox equivalent antioxidant capacity.

### 2.5. Total Flavonoid Content (TFC) Assay

The total flavonoid content in the crude extract was determined following the method described by [29]. The principle is based on the formation of a yellow complex between flavonoids and aluminum ions under alkaline conditions. This complex has a characteristic absorption at 510 nm, and its absorbance shows a linear relationship with flavonoid concentration within a specific range, enabling quantification. Briefly, the reaction mixture contained 0.05 mL of crude extract (10 mg/mL), 0.25 mL of double-distilled water, and 15 μL of 5% sodium nitrite solution. After 8 min, 30 μL of 10% aluminum chloride hexahydrate solution was added. After another 5 min, 0.1 mL of 1 M sodium hydroxide solution and 55 μL of deionized water were added, and the solution was mixed thoroughly. The absorbance of the reaction mixture was immediately measured at 510 nm. Total flavonoid content was calculated using rutin as the standard, based on its standard curve (*y* = 1.4297*x* + 0.00005; *R*^2^ = 0.9988). Absorbance data used for calculations were obtained from metabolite samples of fruit tissues from the four treatments. The final results are expressed as milligrams of rutin equivalent per gram of dry weight of *E. macrophylla* fruit tissue.

### 2.6. Determination of Total Phenolic Content (TPC)

TPC was determined using the Folin–Ciocalteu colorimetric method as previously described [30]. This method involves the reduction of the Folin-Ciocalteu reagent (phosphomolybdate–phosphotungstate) by phenolic compounds in an alkaline medium, forming a stable blue complex. This product has a maximum absorption at 760 nm, and the absorbance is linearly related to phenol concentration, enabling quantification. Specifically, 10 μL of the sample (10 mg/mL) was added to a tube containing 465 μL of 2% sodium carbonate solution. After mixing and standing at room temperature for 2 min, 25 μL of Folin–Ciocalteu reagent was added. The reaction system was then incubated at 40 °C for 2 h. Subsequently, 200 μL of the reaction mixture was transferred to a microplate, and the absorbance was measured at 760 nm. A standard curve was plotted using gallic acid as the positive control (*y* = 1.8231*x* + 0.0921, *R*^2^ = 0.9962), and the total phenolic content in the samples was calculated accordingly. The content was calculated based on the absorbance values from fruit metabolite samples across the four periods and is expressed as milligrams of gallic acid equivalent per gram of dry weight of *E. macrophylla* fruit tissue.

### 2.7. ABTS and DPPH Radical Scavenging Activity Assays

The ABTS radical scavenging activity was determined according to a previous method with slight modifications [31]. Briefly, ABTS was dissolved in distilled water to a concentration of 7 mM, and potassium persulfate was added to a final concentration of 2.45 mM. The mixture was incubated in the dark at room temperature for 12–16 h to generate ABTS^+^ radicals. The ABTS^+^ solution was then diluted with phosphate-buffered saline (PBS, pH 7.4) to an absorbance of 0.70 ± 0.02 at 734 nm. Sample extracts (20 μL) were mixed with 180 μL of diluted ABTS^+^ solution and incubated for 6 min at room temperature. The absorbance was measured at 734 nm using a microplate reader. Trolox was used as a standard, and the results are expressed as μmol Trolox equivalent (TE) per gram of fresh weight.

The DPPH radical scavenging activity was measured following a previously described method with minor modifications [32]. Briefly, 100 μL of sample extract was mixed with 100 μL of 0.2 mM DPPH solution (prepared in 95% ethanol). The mixture was then incubated in the dark at room temperature for 30 min, and the absorbance was measured at 517 nm using a microplate reader (Thermo Fisher Scientific, Waltham, MA, USA). Trolox was used as a standard, and the results were expressed as μmol Trolox equivalent (TE) per gram of fresh weight.

### 2.8. Determination of Total Anthocyanin Content (TANC)

TANC was determined using the pH differential method, with specific steps referenced and slightly optimized from [30]. Briefly, 0.5–1.0 g of the sample was weighed and extracted with methanol containing 0.1% hydrochloric acid via ultrasonication for 30 min, followed by centrifugation at 4000 r/min for 10 min. The supernatant was diluted to 25 mL, and aliquots of the extract were separately mixed with pH 1.0 buffer (0.025 mol/L potassium chloride) and pH 4.5 buffer (0.4 mol/L sodium acetate). Using the respective buffers as blanks, absorbance was measured at 510 nm and 700 nm. The absorbance difference (ΔA) was calculated using the formula ΔA = [(A_510_ − A_700_) at pH 1.0] − [(A_510_ − A_700_) at pH 4.5]. Total anthocyanin content was calculated using cyanidin-3-O-glucoside as the standard, with the following formula: total anthocyanin content (mg/g) = (ΔA × V × 449.2)/(26,900 × 1 × m), where V is the final volume (L), 26,900 L/(mol·cm) is the molar absorptivity of cyanidin-3-O-glucoside, 449.2 g/mol is its molar mass, the cuvette path length is 1 cm, and m is the sample mass (g). All operations were performed under light protection and below 25 °C.

### 2.9. Determination of Total Alkaloid Content (TAC)

TAC was determined using ultrasound-assisted extraction combined with spectrophotometry [33]. The sample was dried at 60 °C to a constant weight and ground to pass through a 60-mesh sieve. Precisely 0.05 g of the powder was weighed into a 2 mL centrifuge tube, and 1 mL of extraction solvent (dichloromethane–methanol–0.5% hydrochloric acid solution, 40:10:1 by volume) was added. After extraction by shaking at room temperature for 30 min, ultrasound-assisted extraction was performed for 30 min (program: 3 min ultrasonication, 1 min shaking, cycled). After extraction, the volume was adjusted to 1 mL with the same solvent, the solution was centrifuged at 4000 r/min for 10 min, and the supernatant was taken for absorbance measurement at 415 nm.

### 2.10. Determination of Total Terpenoid Content (TTC)

TTC was determined following previously reported methods [34]. Precisely 0.1 g of freeze-dried fruit powder from the composite sample of each biological replicate was weighed, and 1.5 mL of anhydrous ethanol was added. Ultrasonic extraction was performed at 40 °C and 300 W for 40 min (with shaking every 10 min). The extract was then centrifuged at 8000× *g* for 10 min, and the supernatant was collected. The residue was extracted twice more and the combined supernatants were diluted to 10 mL to obtain the crude terpenoid extract. Quantification was performed using the vanillin–perchloric acid colorimetric method, in which 0.2 mL of the crude extract was mixed with 0.8 mL of anhydrous ethanol, 0.5 mL of 5% vanillin–glacial acetic acid solution, and 0.5 mL of perchloric acid. The mixture was reacted in a 60 °C water bath for 15 min, cooled in an ice bath, and then mixed with 5 mL of glacial acetic acid. Absorbance was measured at 550 nm. A standard curve was plotted using linalool as the standard (concentration range 0.02–0.10 mg/mL, *R*^2^ ≥ 0.999), and the total terpenoid content in the sample was calculated based on the curve. Wuhan Normickoda Biotechnology Co., Ltd., completed this part of the experiment.

### 2.11. Metabolite Profiling and Statistical Analysis

Metabolite analysis was performed using a UPLC-MS/MS-based, widely targeted metabolomics approach, with the specific protocol referenced and optimized from the published literature [35]. For metabolomics analysis, one composite sample per biological replicate (tray) was prepared as follows. The frozen composite fruit samples from each tray were de-stoned, and the resulting flesh tissue was freeze-dried using a vacuum freeze-dryer (Scientz-100F; Ningbo Scientz Biotechnology Co., Ltd., Ningbo, China). After complete lyophilization, the tissue was ground into a fine powder using a grinding mill (MM 400; Retsch, Haan, North Rhine-Westphalia, Germany) with zirconia beads at 30 Hz for 1.5 min. The powder from all 30 fruits within each tray were thoroughly homogenized to produce a single uniform powder per biological replicate. Exactly 50.00 mg of the freeze-dried powder from each biological replicate was weighed into a 2 mL centrifuge tube for metabolite extraction. Each biological replicate was therefore represented by a single metabolomics sample. Next, 1200 μL of a 70% methanol aqueous solution (*v*/*v*), pre-cooled to −20 °C, was added. The mixture was vigorously vortexed for 30 s, repeated six times, to ensure thorough metabolite extraction. Subsequently, it was centrifuged at 12,000× *g* for 10 min at 4 °C, and the supernatant was carefully collected. Finally, the supernatant was filtered through a 0.22 μm microporous membrane (SCAA-104, ANPEL Laboratory Technologies, Shanghai, China), and the filtrate was collected in vials for subsequent ultra-performance liquid chromatography–tandem mass spectrometry (UPLC-MS/MS) analysis.

Quality control (QC) samples were prepared by pooling equal aliquots from all individual samples. These pooled QC samples were used for three purposes: (i) conditioning the chromatographic column prior to the analytical run; (ii) monitoring system stability and evaluating measurement precision by injecting QC samples at the beginning and end of each sequence and after every 10 experimental samples; and (iii) performing signal correction for analytical drift during data processing [36].

Chromatographic separation was performed on an ultra-high-performance liquid chromatography system (ExionLC™ AD, SCIEX, Redwood City, CA, USA). Separation was achieved using an Agilent (Agilent Technologies, Santa Clara, CA, USA) SB-C18 column (1.8 μm, 2.1 mm × 100 mm) maintained at 40 °C, with an injection volume of 2 μL. The mobile phase consisted of water with 0.1% formic acid (A) and acetonitrile with 0.1% formic acid (B). The elution gradient was set as follows: 0–9 min, 5% B linearly increased to 95% B; 9–10 min, maintained at 95% B; 10–11 min, 95% B rapidly decreased to 5% B; 11–14 min, equilibrated at 5% B. The flow rate was constant at 0.35 mL/min. Mass spectrometric detection was performed using an electrospray ionization triple quadrupole linear ion trap mass spectrometer (QTRAP^®^ 6500+, SCIEX, Redwood City, CA, USA), with data acquired in positive and negative ion modes separately. Ion source parameters were set as follows: ion source temperature, 550 °C; ion spray voltage, +5500 V (positive mode)/−4500 V (negative mode); nebulizer gas (GS I), 50 psi; heater gas (GS II), 60 psi; curtain gas (CUR), 25 psi. The collision gas was nitrogen, set to medium. Data acquisition was performed in Multiple Reaction Monitoring (MRM) mode, with declustering potential and collision energy optimized for each target metabolite.

Metabolite annotation was performed following a tiered confidence system based on the Metabolomics Standards Initiative (MSI) guidelines [37]. Metabolites were annotated based on MS1 accurate mass and MS/MS spectral matching against an in-house spectral library established by Chen et al. [38] and public databases. According to MSI guidelines, these metabolites were classified as Level 2 (putatively annotated compounds), as authentic standards were not available for all detected compounds. Throughout this manuscript, all reported metabolites should be understood as putatively annotated rather than definitively identified, and metabolite names are used for descriptive purposes to facilitate discussion [37].

For data processing, peak integration and quality filtering were performed using the instrument’s proprietary software (SCIEX OS, version 3.0, SCIEX, Redwood City, CA, USA). Metabolite features with a coefficient of variation (CV) > 30% in QC samples were excluded to ensure analytical reproducibility. Missing values were handled using the k-nearest neighbors (KNN) imputation method, implemented through the MetwareBio software suite (https://cloud.metware.cn/). Data normalization was performed using the Probabilistic Quotient Normalization (PQN) method, which corrects for systematic variations arising from sample preparation differences and instrumental drift [39]. Internal standards (six compounds spiked prior to extraction) were used to monitor extraction efficiency and injection reproducibility, and their relative standard deviations (RSDs) were consistently below 15% across all analytical runs [40].

To identify differentially accumulated metabolites (DAMs) between comparison groups, orthogonal partial least squares discriminant analysis (OPLS-DA) was first performed using SIMCA 14.1 software (Umetrics, Umeå, Sweden). The variable importance in projection (VIP) values were extracted from the OPLS-DA model, and metabolites with VIP ≥ 1 were retained for further screening [41]. The OPLS-DA models were validated by seven-fold cross-validation, with model quality assessed using the cumulative explained variance (R^2^Y) and predictive ability (Q^2^). To prevent overfitting, 200 permutation tests were performed for each comparison; models with Q^2^ > 0.5 and a significant difference between the permuted and original R^2^ and Q^2^ values were considered valid.

Subsequently, DAMs were screened using the following combined criteria: |log_2_FC| ≥ 1, VIP ≥ 1, and statistical significance (*p* < 0.05) as determined by paired Student’s *t*-tests, which explicitly account for the repeated-measures design of sampling the same biological replicates across time points. False discovery rate (FDR) correction was applied for multiple comparisons. Principal component analysis (PCA) was performed on the metabolite data from all samples using SIMCA 14.1 software to assess inter-group separation trends and overall variation patterns [42].

### 2.12. Kyoto Encyclopedia of Genes and Genomes (KEGG) Pathway Enrichment Analysis

To further characterize the putatively annotated metabolites and map them to specific biological pathways, the KEGG Compound database (http://www.kegg.jp/kegg/compound/) (accessed on 18 January 2026) was employed for functional annotation [43]. Subsequently, the annotated metabolites were mapped to the KEGG Pathway database. KEGG pathway enrichment analysis was performed using a hypergeometric test to evaluate whether DAMs were over-represented in specific pathways. The resulting *p*-values were adjusted for multiple comparisons using the false discovery rate (FDR) method, and pathways with a corrected *p* < 0.05 were considered significantly enriched.

### 2.13. Validation of DAMs by Quantification Analysis

To ensure the reliability of the metabolomics screening results, an external standard method was employed for the precise quantification of selected differentially accumulated metabolites, following a previously described protocol [44]. This was performed to convert the relative quantitative data from metabolomics analysis into absolute quantitative results with defined concentrations, thereby verifying the accuracy and reproducibility of the metabolite changes. Purified standards were purchased from Sigma-Aldrich. For standard curve construction, standards were dissolved in methanol solvent and stored at 4 °C. The quantitative concentration range for the target analytes in the standards was set from 0.025 to 0.250 mg/mL. All experimental analyses were performed with three biological replicates, with each replicate represented by a single composite sample.

### 2.14. Statistical Analysis

All physiological and biochemical data (including enzyme activities, primary and secondary metabolite contents, and antioxidant activities) were analyzed using SPSS 26.0 software (IBM Corp., Armonk, NY, USA). Data are expressed as means ± standard error (SE) from three independent biological replicates (*n* = 3), with each replicate consisting of a separate storage tray sampled repeatedly at T0, T4, T8, and T12.

Given that the same biological replicates were measured across all four time points, a repeated-measures one-way analysis of variance (RM-ANOVA) was performed to account for the correlated structure of the data arising from repeated sampling of the same experimental units. When a significant overall effect was detected (*p* < 0.05), Tukey’s honestly significant difference (HSD) post hoc test was applied for pairwise comparisons among the four time points. This awareness of the repeated-measures design was applied consistently across all statistical analyses in this study; accordingly, all pairwise comparisons of metabolite abundances were performed using paired tests (see Section 2.11). Statistical significance levels were set at * *p* < 0.05, ** *p* < 0.01, and *** *p* < 0.001. All statistical graphs were generated using GraphPad Prism 9.0 (GraphPad Software, San Diego, CA, USA).

## 3. Results

### 3.1. Morphological and Visual Changes During Storage

**Figure 1:**
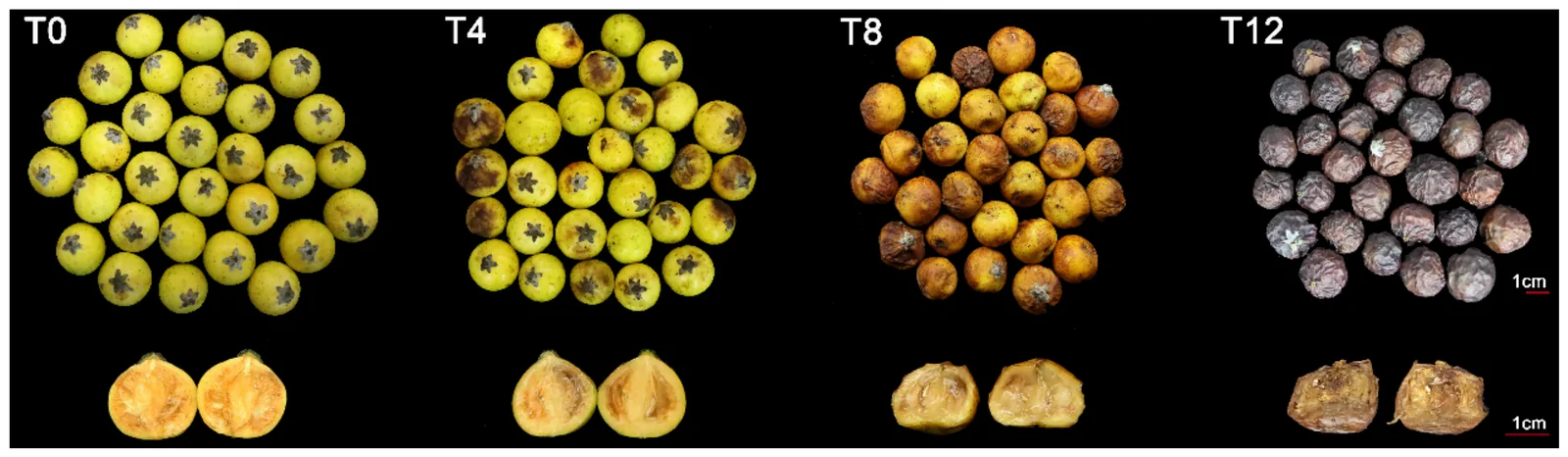
Changes in the external morphology of *E. macrophylla* fruits during post-harvest storage. Images show representative fruits sampled at T0 (day 0, fresh control), T4 (day 4), T8 (day 8), and T12 (day 12).

During ambient storage, the external morphology of *E. macrophylla* fruits underwent marked changes (Figure 1). At T0 (day 0), fruits exhibited intact morphology with a yellowish-green peel and white flesh, with the flesh browning rapidly upon cutting. By T4 (day 4), approximately 30% of fruits showed peel browning, while flesh browning remained mild. At T8 (day 8), all fruits displayed complete peel browning, accompanied by intensified flesh browning and visible shriveling due to water loss. By T12 (day 12), shriveling further increased, and both peel and flesh turned fully brown. These observations document a progressive decline in visual quality, characterized by advancing browning and dehydration.

### 3.2. Dynamic Changes in Primary and Secondary Metabolites and Antioxidant Activity

**Figure 2:**
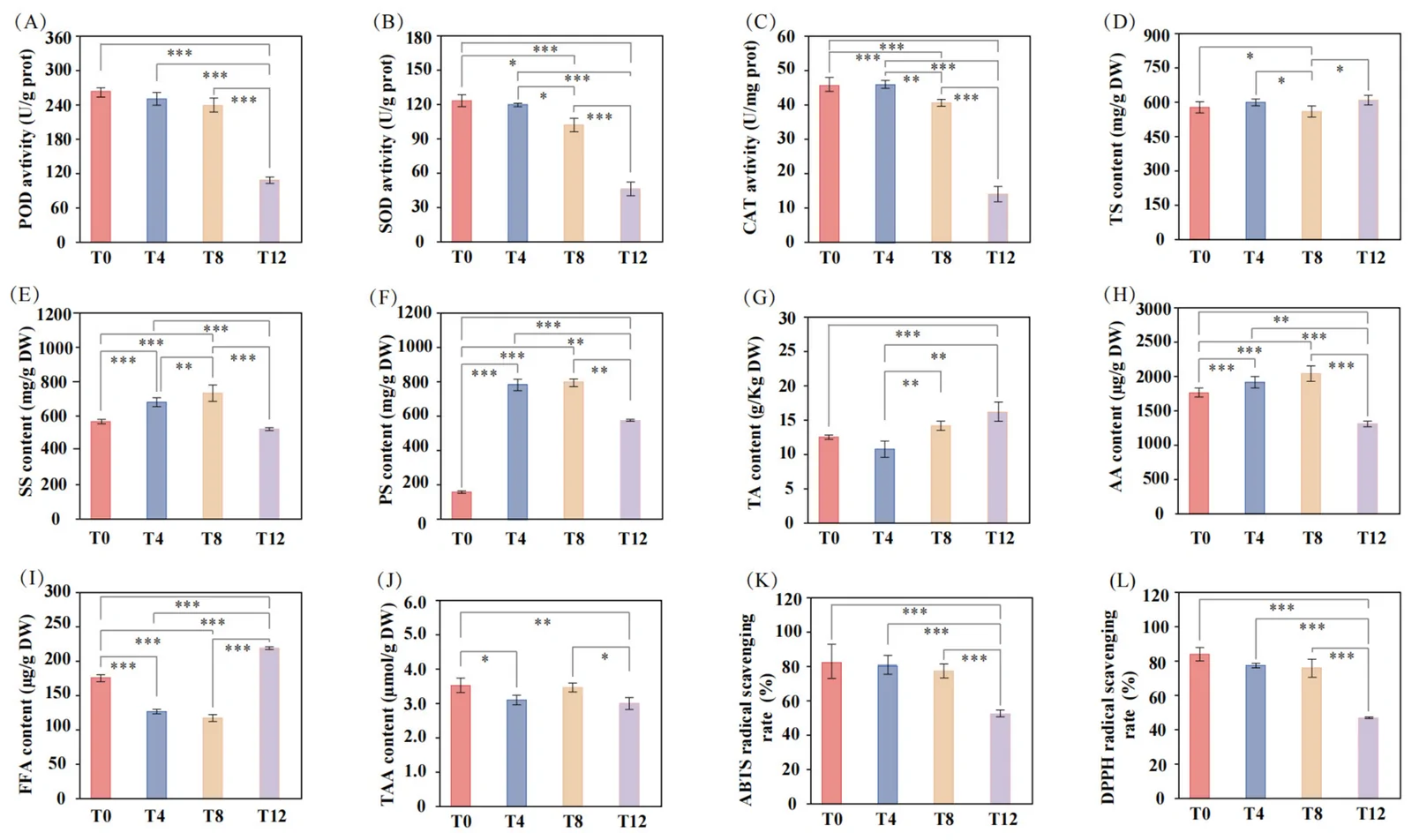
Changes in physiological parameters and bioactive compound contents of *E. macrophylla* fruits during storage. (**A**) Peroxidase (POD) activity. (**B**) Superoxide dismutase (SOD). (**C**) Catalase (CAT) activity. (**D**) Total sugar (TS) content. (**E**) Soluble sugar (SS) content. (**F**) Polysaccharide (PS) content. (**G**) Total acid (TA) content. (**H**) Ascorbic acid (AA) content. (**I**) Free fatty acid (FFA) content. (**J**) Total amino acid (TAA) content. (**K**) ABTS radical scavenging activity. (**L**) DPPH radical scavenging activity. Data are presented as mean ± standard error (*n* = 3). Asterisks indicate significant differences among storage time points (* *p* < 0.05, ** *p* < 0.01, *** *p* < 0.001).

Parallel to morphological deterioration, substantial shifts in internal physiological and biochemical profiles were detected (Figure 2). The activities of antioxidant enzymes—peroxidase (POD), superoxide dismutase (SOD), and catalase (CAT)—were significantly lower at T12 than at T0, T4, or T8 (Figure 2A–C). Total sugar (TS) content remained largely unchanged throughout storage (Figure 2D). In contrast, soluble sugar (SS) and polysaccharide (PS) contents first increased and then decreased (Figure 2E,F), whereas total acid (TA) rose progressively (Figure 2G). Ascorbic acid (AA) increased initially before declining (Figure 2H), and free fatty acid (FFA) content decreased first and later increased (Figure 2I). The total amino acid (TAA) content fell continuously, with the highest level at T0 and the lowest at T12 (Figure 2J). Accordingly, the antioxidant capacity, assessed using ABTS and DPPH radical scavenging assays, dropped significantly by T12 (Figure 2K,L).

**Figure 3:**
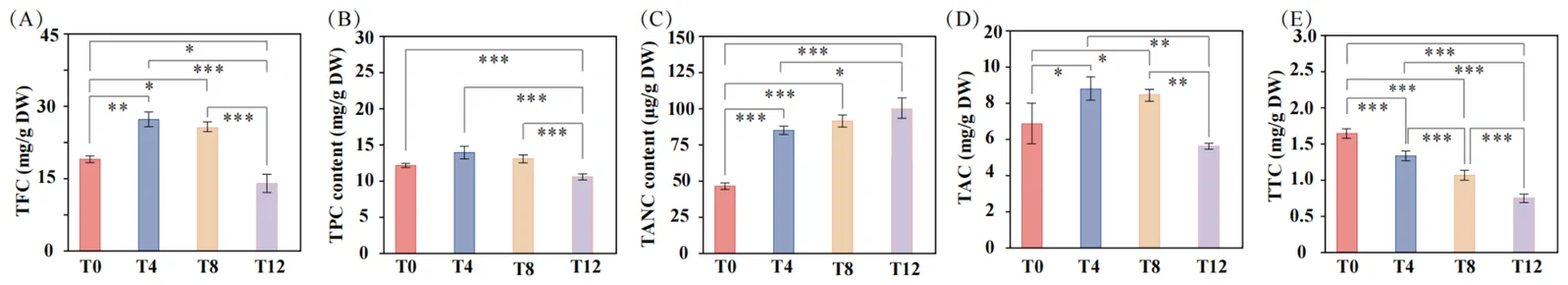
Changes in secondary metabolite contents of *E. macrophylla* fruits during storage. (**A**) Total flavonoid content (TFC). (**B**) Total phenolic content (TPC). (**C**) Total anthocyanin content (TANC). (**D**) Total alkaloid content (TAC). (**E**) Total terpenoid content (TTC). Data are presented as mean ± standard error (*n* = 3). Asterisks indicate significant differences among storage time points (* *p* < 0.05, ** *p* < 0.01, *** *p* < 0.001).

Changes in secondary metabolites followed distinct dynamics (Figure 3). Total flavonoid (TFC), total phenolic (TPC), and total alkaloid (TAC) contents first rose, peaked at mid-storage, and then fell to minima at T12 (Figure 3A,B,D). Total terpenoid (TTC) content declined progressively throughout storage (Figure 3E), while total anthocyanin (TANC) content increased steadily, reaching its maximum at T12 (Figure 3C). The overall reduction in antioxidant activity during storage coincided with the decline in total phenolics, flavonoids, and alkaloids, consistent with a positive association between these metabolite pools and the fruit’s antioxidant capacity.

### 3.3. Metabolome Profiling in E. macrophylla Fruits During Storage

**Figure 4:**
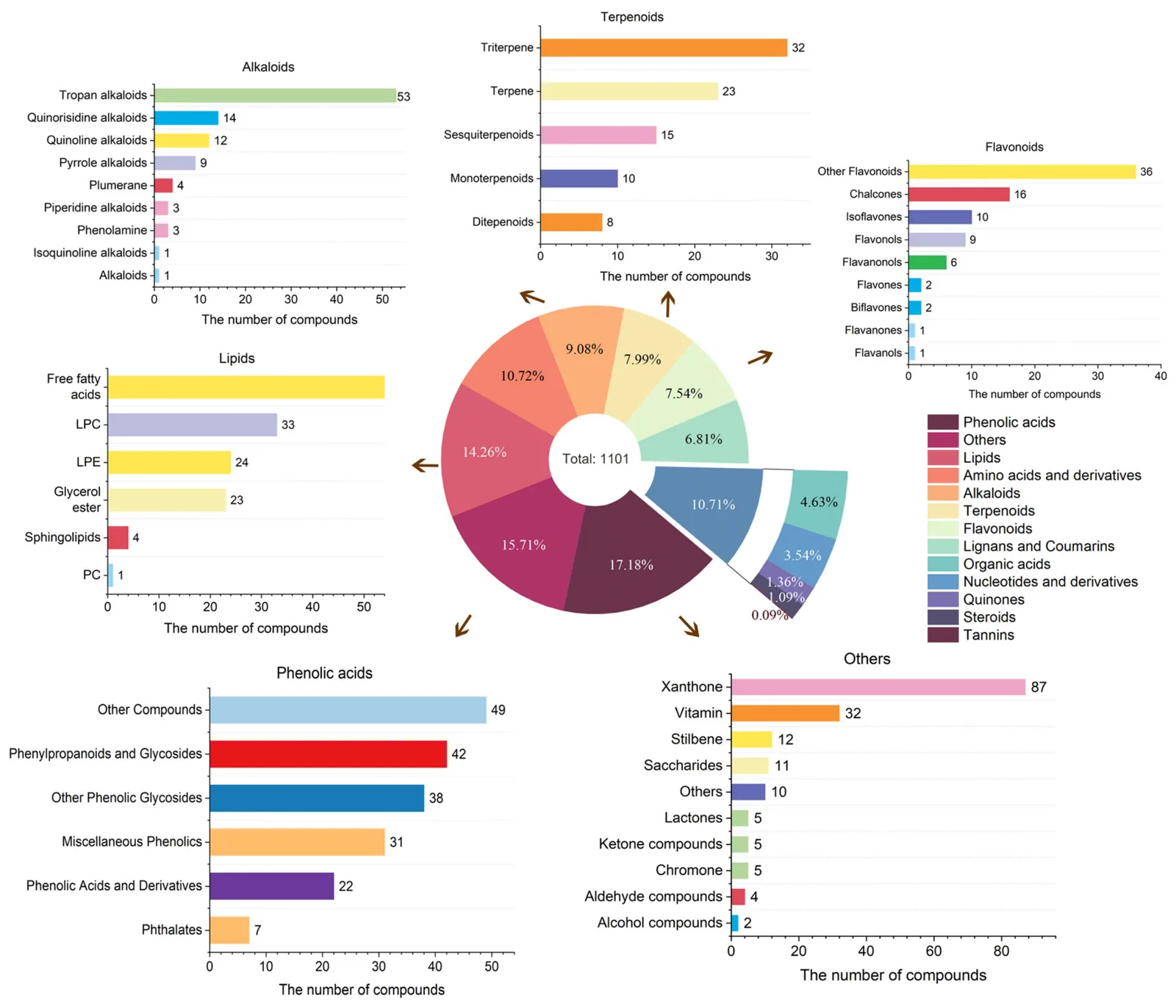
Annotation and classification of metabolites in *E. macrophylla* fruits. A total of 1101 metabolites were putatively annotated and categorized into 13 classes.

To characterize the global metabolite profiles, a widely targeted metabolomics analysis was performed using UPLC-MS/MS. A total of 1101 metabolites were putatively annotated and relatively quantified across all samples (Figure 4), and these metabolites were classified into 13 major categories, with phenolic acids (189), lipids (157), and amino acids and derivatives (118) being the most abundant, followed by alkaloids (100), terpenoids (88), flavonoids (83), lignans and coumarins (75), organic acids (51), nucleotides and derivatives (39), quinones (15), steroids (12), and tannins (1). The remaining 173 metabolites were grouped as “others” (Table S2).

**Figure 5:**
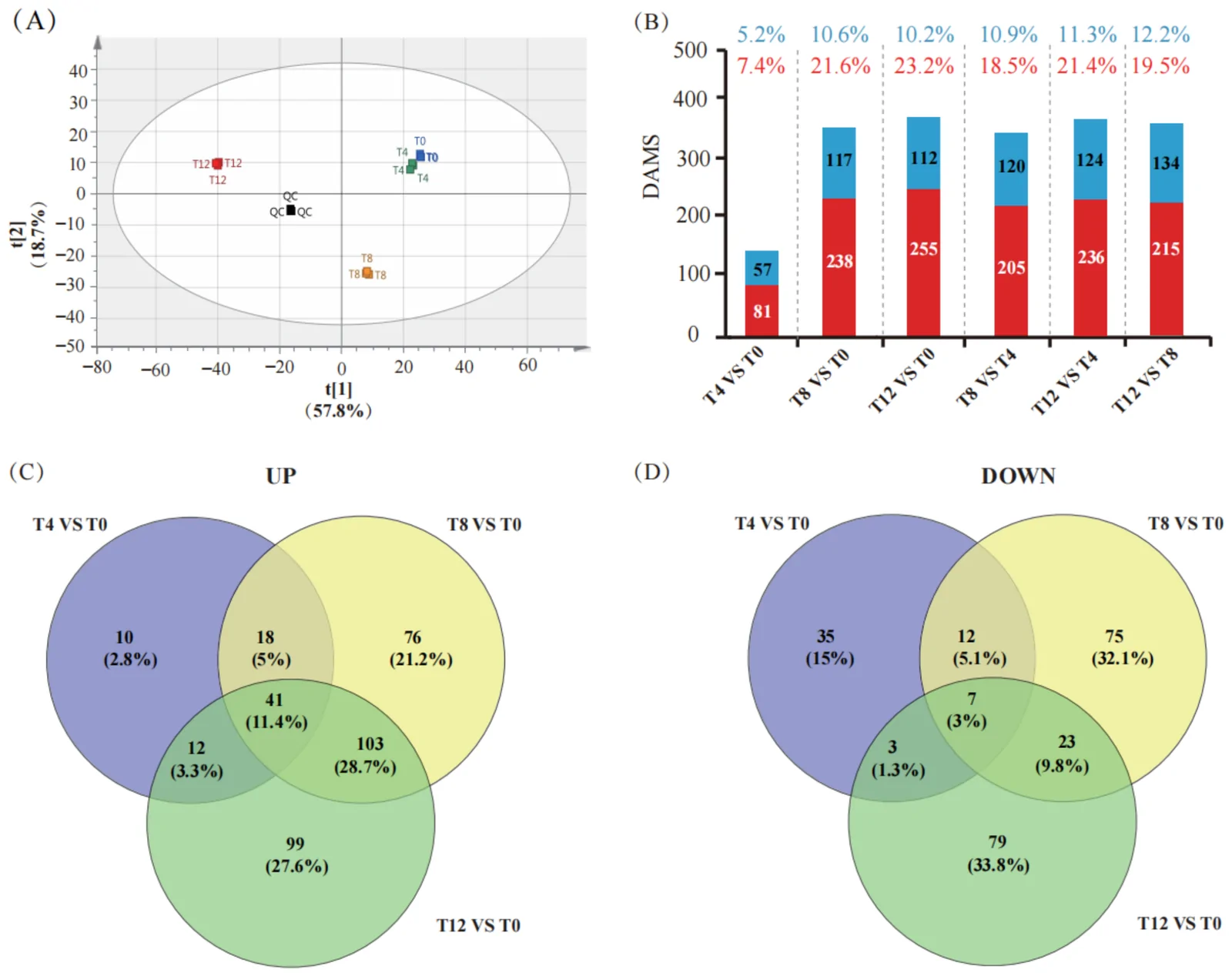
Comprehensive analysis of metabolomic profiles in *E. macrophylla* fruits across different storage time points. (**A**) Principal component analysis (PCA) of the metabolomes. The x-axis (PC1) and y-axis (PC2) show the scores, illustrating the separation of samples from different storage periods (T0, T4, T8, T12). (**B**) Number of differentially accumulated metabolites (DAMs) in pairwise comparisons among all four time points. Red and blue bars represent up-regulated and down-regulated metabolites, respectively. (**C**,**D**) Venn diagrams showing the shared and unique metabolites that were up-regulated (**C**) or down-regulated (**D**) at each later storage point (T4, T8, T12) compared to the initial time point (T0).

Principal component analysis (PCA) revealed a clear separation among the four storage time points (T0, T4, T8, T12) in multivariate space, with samples from the same time clustering tightly together (Figure 5A). T4 samples remained relatively close to T0 along both principal components, whereas T8 and T12 samples were markedly separated from earlier time points, indicating a substantial metabolic shift after eight days of storage. The first principal component (PC1), accounting for 57.8% of the total variance, primarily separated T12 from the other groups. The second component (PC2), explaining 18.7% of the variance, distinguished T8 from the remaining time points (Figure 5A).

Pairwise comparisons of differentially accumulated metabolites (DAMs) across all four time points showed that the number of DAMs between T4 and T0 was the lowest among all comparisons (Figure 5B; Tables S3–S8). In contrast, all other pairwise comparisons—including T8 vs. T0, T12 vs. T0, T8 vs. T4, T12 vs. T4, and T12 vs. T8—exhibited a substantial increase in DAMs, accounting for 30–40% of the total metabolites detected. Notably, in every comparison where a later storage point was evaluated against an earlier one, the number of up-regulated DAMs significantly exceeded that of down-regulated DAMs. Venn analysis highlighted the specificity and overlap of metabolic changes over time (Figure 5C,D). Among up-regulated DAMs, only 41 (11.4% of total up-regulated) were common to T4, T8, and T12 when compared to T0. In contrast, 76 (21.2%) and 99 (27.6%) metabolites were specifically up-regulated in T8 and T12, respectively. For down-regulated DAMs, the overlap was even smaller, with only seven metabolites (3.0% of total down-regulated) commonly decreased across all three later time points, while T4, T8, and T12 exhibited 35 (15.0%), 75 (32.1%), and 79 (33.8%) uniquely down-regulated metabolites, respectively. These results collectively indicate that post-harvest storage induces a progressive and extensive metabolic reprogramming in *E. macrophylla* fruits, with the transition at T8 representing a pronounced turning point.

To validate the metabolomic findings, six representative DAMs spanning distinct chemical classes, including flavonoid glycosides (hesperetin 5-O-glucoside, apigenin 5-O-glucoside, kaempferol 3-O-glucoside), a phenolic acid (cinnamic acid), a phenolamine (N-Feruloylputrescine), and an amino acid derivative (Glu-Phe), were selected for targeted quantification using external standards. Pearson correlation analysis was performed to evaluate the concordance between log_2_(fold-change) values derived from untargeted metabolomics and those obtained from targeted absolute quantification. The analysis included measurements from four time points (T0, T4, T8, and T12), with three biological replicates per time point, yielding 12 paired observations (4 time points × 3 replicates) per metabolite. A high degree of concordance (R^2^ > 0.80) was observed between the two datasets (Figure S1). This high concordance across multiple time points and the diverse metabolite classes confirm the reliability of the widely targeted metabolomics approach in capturing dynamic metabolite changes during fruit storage.

### 3.4. Identification of Differential Metabolites During Post-Harvest Storage

**Figure 6:**
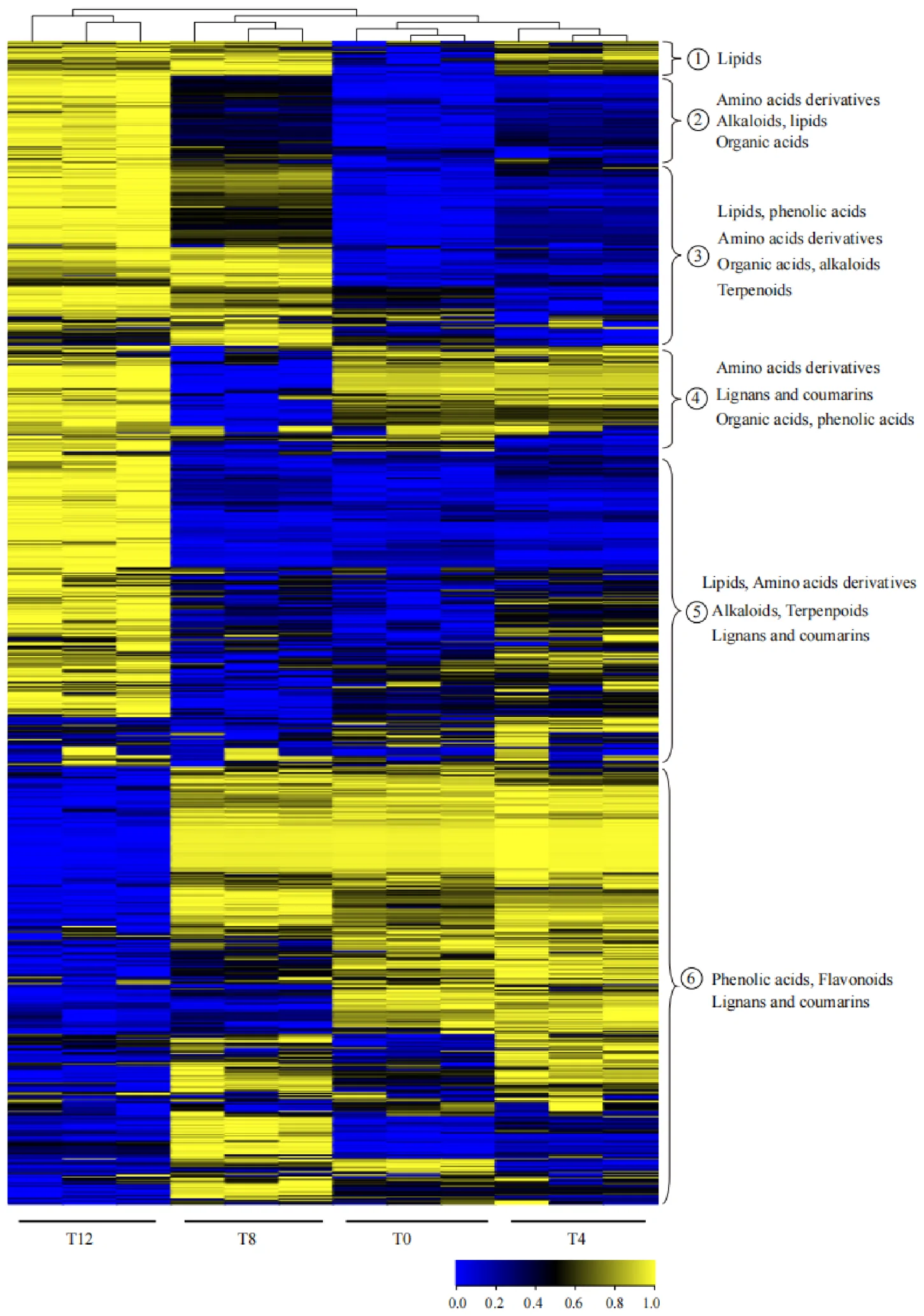
Hierarchical clustering heatmap of differentially accumulated metabolites in *E. macrophylla* fruits during postharvest storage. Rows represent individual metabolites, and columns represent the four storage time points (T0, T4, T8, T12). Metabolites are clustered into distinct groups (1–6, labeled on the right) based on their accumulation patterns. The color scale indicates relative metabolite abundance (red for higher, green for lower). Major enriched metabolite classes within each cluster are annotated next to the corresponding cluster.

To elucidate the temporal dynamics of the fruit metabolome, differentially accumulated metabolites were analyzed through complementary clustering approaches. Hierarchical clustering revealed six major accumulation patterns (Patterns 1–6; Figure 6). Patterns 1, 2, and 5 showed a progressive increase, peaking at T12, and were enriched in lipids, amino acid derivatives, and organic acids. Pattern 3 exhibited delayed accumulation, rising sharply from T8 to T12, and was associated with a broad profile that included phenolic acids. In contrast, Pattern 4 declined to a trough at T8, featuring amino acid derivatives, lignans, and coumarins, while Pattern 6 (phenolic acids and flavonoids) decreased steadily after T4, reaching a minimum at T12.

**Figure 7:**
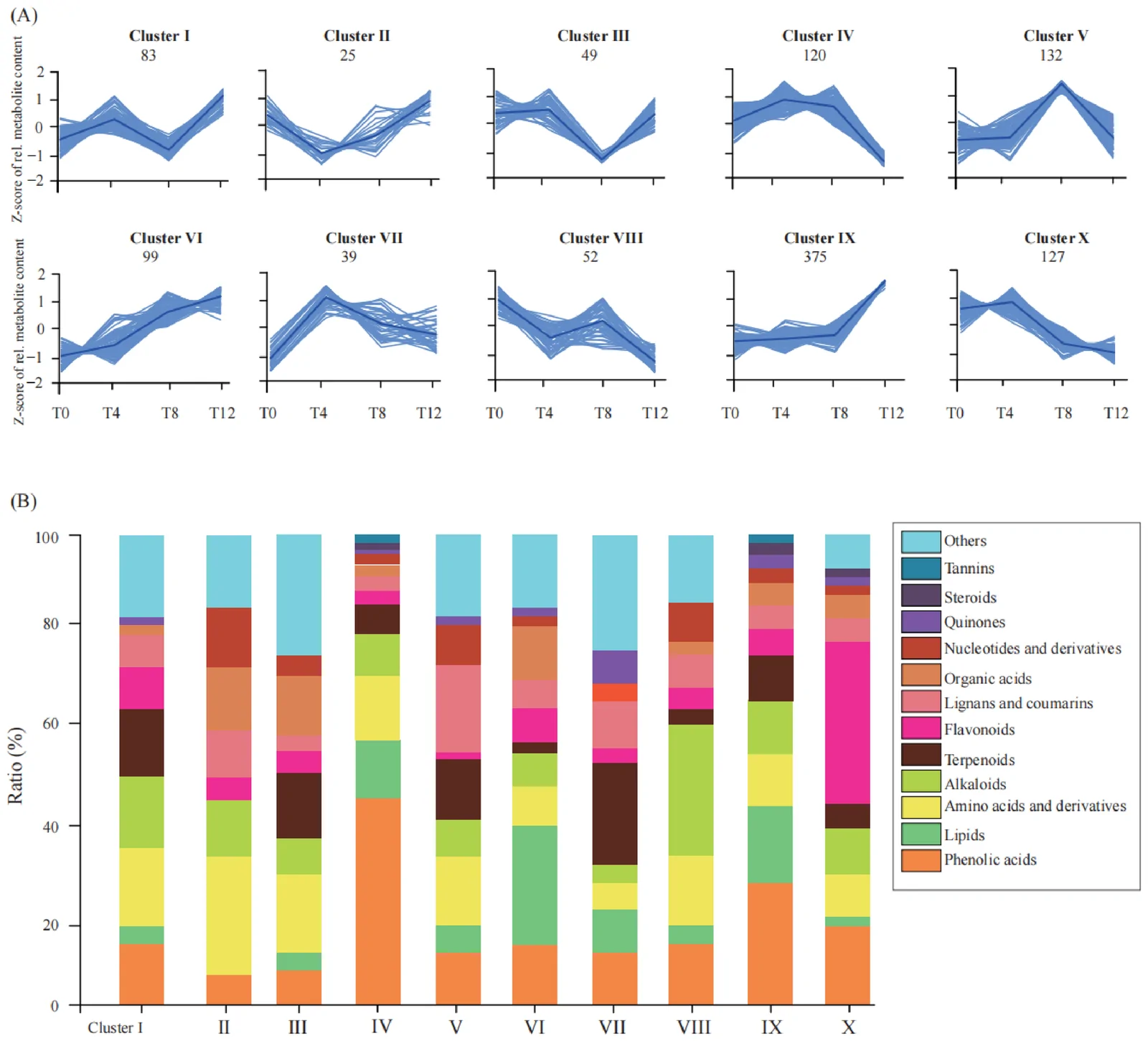
K-means trend clustering analysis of differentially accumulated metabolites in *E. macrophylla* fruits during postharvest storage. (**A**) Temporal accumulation trends of clustered metabolites. The x-axis represents storage time points (T0, T4, T8, T12), and the y-axis represents standardized relative abundance. Each line corresponds to a metabolite cluster (Clusters I–X), with the thick blue line indicating the mean expression trend of each cluster. (**B**) Composition and proportion of major metabolite classes within each cluster.

For finer-grained resolution, *K*-means clustering identified ten sub-clusters (I–X) with distinct compositional profiles (Figure 7; Table S9). Consistent with the hierarchical analysis, Clusters VI and IX, which increased strongly by T12, were predominantly composed of lipids and phenolic acids. Conversely, Clusters VIII and X, which declined markedly, were rich in alkaloids, flavonoids, and phenolic acids. Several clusters displayed clear stage-specificity: metabolites in Cluster V peaked at T8 (lignans, coumarins, amino acid derivatives); Clusters VII and VIII were most abundant at T4 and T0, respectively; and Clusters I, VI, and IX were highly enriched at T12. Collectively, these multi-level clustering analyses demonstrate that postharvest metabolic reprogramming in *E. macrophylla* fruit is a highly structured process involving the coordinated, phase-specific induction and repression of distinct metabolite modules.

### 3.5. Pathway Enrichment of DAMs During Post-Harvest Storage

**Figure 8:**
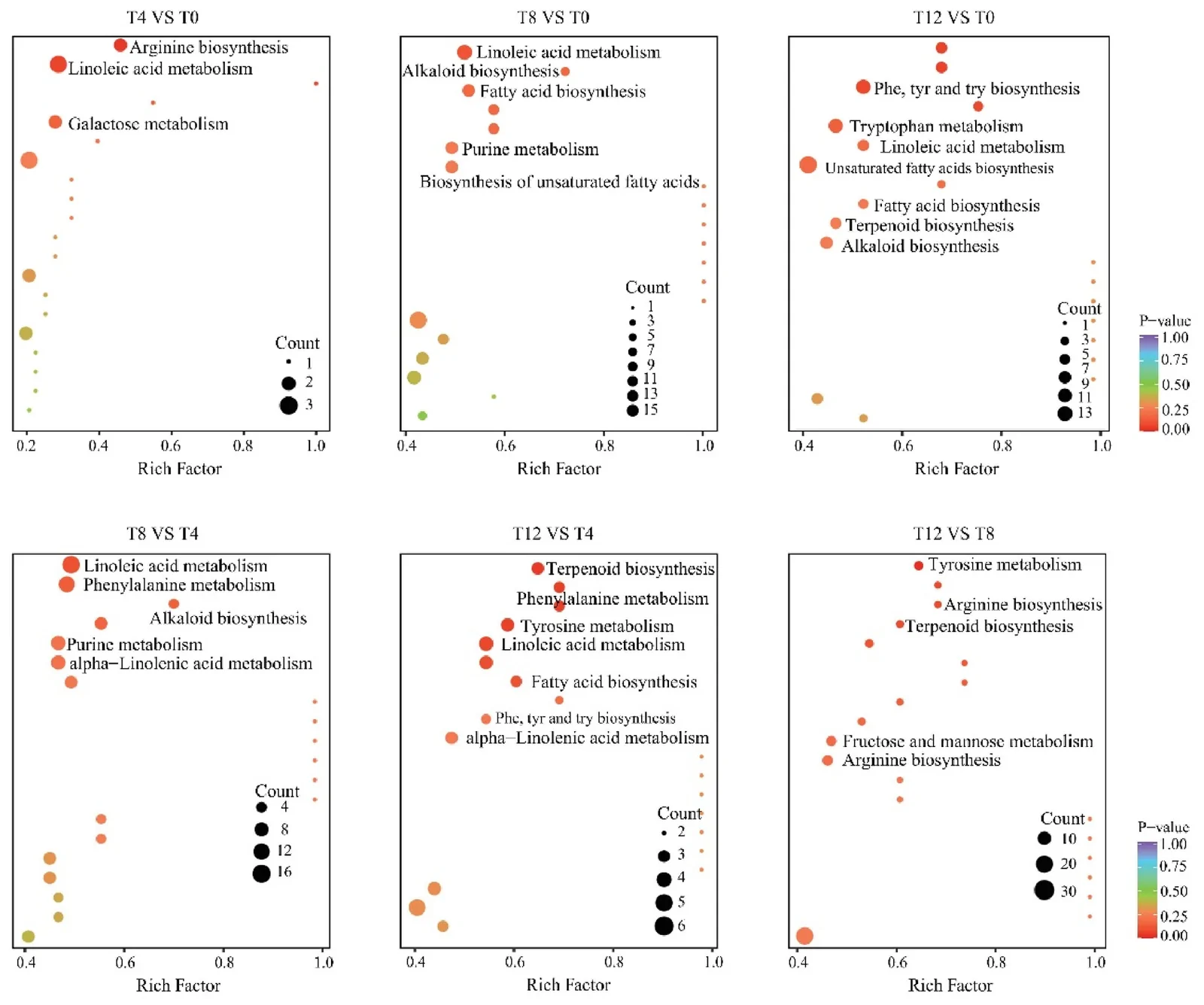
KEGG pathway enrichment analysis of up-regulated differentially accumulated metabolites (DAMs) in *E. macrophylla* fruits during post-harvest storage. Bubble plots display significantly enriched metabolic pathways revealed by pairwise comparisons across all four storage time points (six comparisons in total). For each comparison, only DAMs that were significantly up-regulated in the later time point relative to the earlier time point were subjected to enrichment analysis. The y-axis indicates KEGG pathway names, and the x-axis represents the enrichment factor. Bubble size corresponds to the number of up-regulated DAMs mapped to the respective pathway. Color intensity reflects the statistical significance of enrichment, with a gradient from blue to red.

To elucidate the functional implications of metabolic changes, KEGG pathway enrichment analysis was performed on both up- and down-regulated DAMs from pairwise comparisons across all storage time points (Figure 8 and Figure S2). In the early storage phase (T4 vs. T0), up-regulated DAMs were primarily enriched in pathways related to carbohydrate and lipid metabolism, such as galactose metabolism and linoleic acid metabolism, as well as arginine biosynthesis (Figure 8). Concurrently, down-regulated DAMs at this stage were significantly associated with glycerophospholipid metabolism, terpenoid biosynthesis, and phenylalanine metabolism (Figure S2). As storage progressed to the mid-phase (T8 vs. T0), the spectrum of enriched pathways for up-regulated DAMs expanded notably. Key activated pathways included linoleic acid metabolism, the biosynthesis of unsaturated fatty acids, fatty acid biosynthesis, alkaloid biosynthesis, and purine metabolism (Figure 8). A prominent feature at this and later stages was the consistent enrichment of down-regulated DAMs in the flavone and flavonol biosynthesis pathway (Figure S2). By the late storage phase (T12 vs. T0), up-regulated DAMs showed enrichment in an even broader set of pathways, intensifying involvement in lipid metabolism (unsaturated fatty acid biosynthesis, linoleic acid metabolism, fatty acid biosynthesis), specialized metabolism (terpenoid biosynthesis, alkaloid biosynthesis), and amino acid metabolism (phenylalanine, tyrosine and tryptophan biosynthesis, tryptophan metabolism) (Figure 8). Furthermore, comparisons between consecutive later time points (T8 vs. T4, T12 vs. T4, T12 vs. T8) revealed that up-regulated DAMs continued to be enriched in linoleic acid metabolism, various amino acid synthesis/metabolic processes, and the biosynthesis of terpenoids and alkaloids (Figure 8). In contrast, down-regulated DAMs in these comparisons were persistently linked to flavone and flavonol biosynthesis and the citrate cycle (TCA cycle) (Figure S2).

This temporal analysis reveals a clear reprogramming of metabolic pathways: early storage involves adjustments in primary carbohydrate and lipid metabolism, while mid-to-late storage is characterized by the progressive activation of pathways for fatty acid diversification, alkaloid/terpenoid biosynthesis, and aromatic amino acid metabolism. The sustained lower abundance of metabolites mapped to the flavonoid biosynthesis pathway was consistent with the observed decline in total flavonoid content and antioxidant activity over time.

### 3.6. Temporal Dynamics of Key Metabolites Within Enriched Pathways

**Figure 9:**
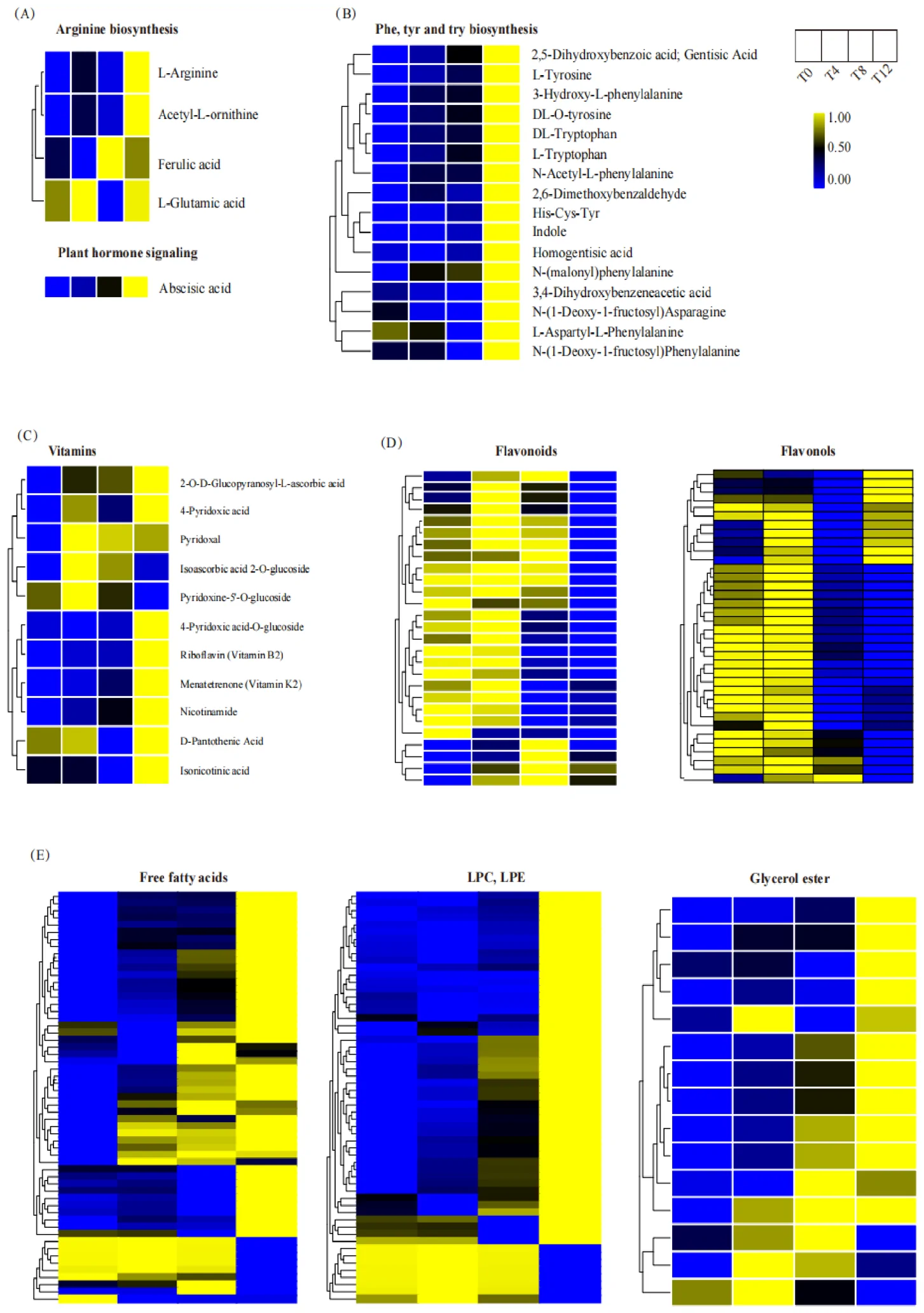
Temporal expression heatmaps of key metabolites within enriched KEGG pathways during post-harvest storage of *E. macrophylla* fruits. The heatmaps depict the standardized relative abundance patterns of representative metabolites across four storage time points (T0, T4, T8, T12). Subpanels correspond to the following significantly enriched pathways or metabolite categories: (**A**) arginine biosynthesis and plant hormone signaling, (**B**) phenylalanine, tyrosine and tryptophan biosynthesis, (**C**) vitamin metabolism, (**D**) flavonoid and flavonol metabolism, and (**E**) lipid metabolism (including free fatty acids, lysophospholipids, and glycerol esters). The color gradient indicates normalized metabolite levels, with yellow representing higher abundance and blue representing lower abundance. Each row represents an individual metabolite, and each column represents a storage time point.

Building on the pathway enrichment results, the expression patterns of representative metabolites within several significantly enriched KEGG pathways were examined across the storage period (Figure 9). This detailed analysis revealed coherent temporal trends within specific metabolic modules. In the arginine biosynthesis pathway (Figure 9A), precursors such as L-glutamic acid and acetyl-L-ornithine showed a marked increase in abundance at T8 and T12, aligning with the sustained enrichment of this pathway in late storage comparisons (Figure 8). Similarly, several metabolites mapped to the phenylalanine, tyrosine, and tryptophan biosynthesis pathway, including various aromatic amino acids and their derivatives, showed a progressive increase, particularly from T8 onward (Figure 9B). The heatmap for vitamin metabolism (Figure 9C) illustrated distinct fates for different vitamins. While compounds like riboflavin (vitamin B_2_) and menatetrenone (vitamin K_2_) increased over time, derivatives of vitamin B_6_ (pyridoxal, 4-pyridoxic acid) showed a declining trend after T4. Metabolites associated with flavonoid and flavonol metabolism, including various flavonols, displayed a general decrease in abundance as storage progressed (Figure 9D), which was consistent with the enrichment of the “Flavone and flavonol biosynthesis” pathway among DAMs with lower abundance in later storage stages (Figure S2). In addition, abscisic acid (ABA), a stress-related plant hormone, also showed a decreasing trend during storage, which may reflect the alleviation of stress responses over time. In contrast, lipid metabolism metabolites (Figure 9E), including various free fatty acids and lysophospholipids (LPC, LPE), exhibited a clear pattern of accumulation, especially in later storage stages (T8, T12). These accumulation patterns are consistent with the enrichment of lipid-related pathways (linoleic acid metabolism, fatty acid biosynthesis) revealed in the KEGG enrichment analysis.

Collectively, the heatmap analysis translates pathway-level enrichment findings into concrete metabolite accumulation trends. These observations are consistent with a relative increase in features associated with amino acid and lipid biosynthesis pathways and a relative decrease in those linked to flavonoid metabolism during prolonged storage, providing a temporal perspective on the potential metabolic reconfiguration occurring in the fruit.

## 4. Discussion

In this study, we characterized the dynamic, stage-specific metabolic changes in *E. macrophylla* fruits during post-harvest storage by integrating physiological measurements, targeted quantification, and widely targeted metabolomics. Our results revealed systematic metabolic remodeling associated with both quality deterioration and the transformation of functional compounds. Critically, based on the findings of this study, we hypothesize that fruit blackening may be associated with the oxidation, polymerization, and degradation of phenolic compounds. However, this hypothesis requires further experimental validation through subsequent studies involving the determination of PPO activity, ROS levels, and polymeric pigment content. Our findings provide a foundation for understanding the material basis of traditionally processed products.

Previous studies on post-harvest fruits have demonstrated that storage-induced quality changes are accompanied by coordinated alterations in primary metabolism, secondary metabolism, antioxidant systems, and stress-responsive pathways [45]. However, most investigations have focused on commercially important fresh fruits, whereas the metabolic trajectory of medicinal–food plants during traditional processing remains comparatively less explored [46]. Unlike fresh fruits undergoing ripening, *E. macrophylla* fruits experience prolonged dehydration and oxidative conditions during storage, which may induce distinct metabolic responses associated with stress adaptation and phytochemical transformation [47].

Metabolic profiling revealed distinct phase transitions during storage, with significant metabolic changes observed at the T8 time point. Several lines of evidence are consistent with this interpretation, including the clear separation of T8 samples in principal component analysis, the increase in differentially accumulated metabolites thereafter, and the phase-specific accumulation patterns revealed by clustering analyses (Figure 5, Figure 6 and Figure 7). This phased response is consistent with previous observations that post-harvest storage can trigger a shift from maintenance metabolism toward stress-associated secondary metabolism. Similar transition periods have been reported in other plant systems. For example, Kim [48] identified days 8 to 9 as a critical stage during strawberry post-harvest storage, characterized by rapid anthocyanin accumulation and condensed tannin degradation, followed by accelerated senescence. Similarly, Li [29] reported that frost stress in *Dioscorea opposita* activated phenylpropanoid, flavonoid, and alkaloid biosynthetic pathways, resulting in a significant accumulation of medicinal metabolites. These studies collectively suggest that post-harvest or environmental stresses may induce time-dependent metabolic reorganization rather than uniform metabolite degradation. In our study, the early phase (T0 to T4) was mainly characterized by initial stress responses and the adjustment of primary metabolites. In contrast, the mid-to-late phase (T8 to T12) involved extensive metabolic redistribution, including enhanced pathways associated with lipid metabolism, alkaloid and terpenoid biosynthesis, and amino acid metabolism (Figure 8). These findings suggest that T8 may represent a critical metabolic inflection point, after which the metabolite profiles are increasingly characterized by features associated with senescence and dehydration. This discovery provides a precise temporal target for interventions aimed at modulating post-harvest quality outcomes.

A central and initially paradoxical finding of our study was the continuous increase in total anthocyanin content accompanied by a general decline in key antioxidant-related compounds, including total phenolics, flavonoids, and alkaloids, together with a reduced free radical scavenging capacity (Figure 2 and Figure 3). This observation indicates that increased pigmentation does not necessarily correspond to enhanced antioxidant potential during post-harvest processing, particularly in plant materials undergoing dehydration and oxidative transformation. A possible explanation for this discrepancy is that prolonged storage may promote the transformation of soluble phenolic compounds. During drying and storage, low-molecular-weight phenolic antioxidants, such as flavonoid glycosides and phenolic acids, may undergo oxidation, enzymatic browning, or polymerization, which could result in changes in their extractability and apparent antioxidant properties [49]. This process not only depletes the extractable pool of bioactive phenolics but also directly drives the visible blackening of the fruit. This mechanism finds robust support in the broader literature on post-harvest fruit browning. In pomegranate peel, Qi [50] reported that total phenolic content gradually decreased during ambient storage, accompanied by enhanced polyphenol oxidase (PPO) activity, increased membrane lipid peroxidation, and elevated malondialdehyde (MDA) levels. These changes suggested that oxidative stress and phenolic oxidation jointly contributed to peel browning. Similarly, in lychee pericarp, Zhang [51] demonstrated that anthocyanin degradation products could generate catechol-like structures, which subsequently promoted PPO-mediated oxidation and accelerated visible browning. Although these studies were mainly conducted in fresh fruits, they provide important insights into the possible oxidative processes occurring during *E. macrophylla* storage. The gradual darkening of *E. macrophylla* fruits may therefore involve not only pigment accumulation but also extensive phenolic conversion and structural modification. In this context, the decline in antioxidant capacity observed in the present study may result from the reduction in extractable phenolic antioxidants and the formation of oxidized phenolic derivatives with different chemical properties. However, phenolic metabolism during post-harvest storage is highly species-dependent. In contrast to browning-sensitive fruits, red-fleshed kiwifruit during ripening exhibits continuous increases in total phenolic content, total flavonoid content, total proanthocyanidin content, and antioxidant capacity, with maximum values observed at the edible ripening stage [52]. This divergence likely stems from kiwifruit’s climacteric nature, where starch degradation provides carbon skeletons for soluble sugars, while ethylene signaling activates phenylpropanoid metabolism, promoting the synthesis of phenolic compounds such as catechins and proanthocyanidins B1 and B2. This comparative perspective underscores that post-harvest phenolic metabolism is highly dependent on species-specific physiological types and the balance between antioxidant defense systems and oxidative stress. Moreover, while anthocyanin dynamics correlate closely with browning progression, their changes, whether during fruit ripening or under stress, may represent both a response to polymerization cascades and an independent biosynthetic response to environmental stress. Therefore, the conventional “total phenolic content” metric may inadequately reflect the actual bioactive antioxidant potential in processed fruits, given the fundamental alterations in the chemical nature and bioavailability of these phenolic compounds. The observed decline in specific flavonoid glycosides in our metabolomics data is consistent with this oxidation–polymerization hypothesis, although direct evidence for the formation of oxidation/polymerization products remains to be obtained.

Beyond phenolic metabolism, our metabolomic analysis revealed extensive alterations in lipid metabolism, amino acid metabolism, and specialized secondary metabolic pathways during prolonged storage. KEGG enrichment analysis and metabolite heatmaps further revealed strategic reprogramming toward the synthesis of specific compound classes (Figure 8 and Figure 9). These metabolic shifts are indicative of a redistribution of metabolic profiles during prolonged storage, with increasing relative abundance of metabolites mapped to stress-associated and secondary metabolic pathways. Lipid metabolism plays an important role in maintaining cellular homeostasis under post-harvest stress conditions. During dehydration and senescence, changes in water availability and oxidative status can affect membrane structure and lipid composition. Plants have been reported to adjust fatty acid metabolism in response to unfavorable conditions [53]. More notably, the enrichment of alkaloid and terpenoid biosynthesis pathways during mid-to-late storage is functionally suggestive. These nitrogen- and isoprenoid-derived compounds are well-established antimicrobial and insect-deterrent agents, and their increased abundance may reflect an adaptive defense response to heightened pathogen susceptibility in senescing, sugar-rich tissues, potentially contributing to fruit storability and traditional medicinal efficacy. Supporting this, Shmuleviz [54] demonstrated that, during post-harvest dehydration storage of wine grapes, terpene synthase and stilbene synthase genes were significantly up-regulated, leading to an increased accumulation of terpenes and the phytoalexin resveratrol, enhancing abiotic stress resistance. In post-harvest mango ripening, the activation of terpenoid biosynthesis pathway genes closely correlates with improved fruit storage quality [55]. Collectively, these studies underscore that post-harvest storage is not merely a continuation of physiological metabolism but a critical phase of dynamic secondary metabolite reprogramming. Different species exhibit highly coordinated molecular adaptations through the specific regulation of terpenoid, stilbenoid, and alkaloid pathways in stress response, quality maintenance, and medicinal transformation. Furthermore, the enrichment of arginine and aromatic amino acid biosynthesis pathways indicates enhanced nitrogen metabolism, potentially supplying precursors for defense compounds. For example, aromatic amino acids provide precursors for phenylpropanoid biosynthesis, while arginine metabolism contributes to nitrogen redistribution and stress signaling regulation [56,57]. The enrichment of metabolites mapped to aromatic amino acid-related pathways observed in this study suggests that the balance of carbon and nitrogen metabolism may shift during late storage, which could be related to adaptive responses. The coordinated regulation of lipid, amino acid, and secondary metabolism observed in this study provides additional evidence that *E. macrophylla* fruits undergo active metabolic remodeling rather than simple chemical degradation during storage. This pattern differs from the metabolic behavior reported for many fresh fruits during ripening, where metabolic changes are mainly associated with improved flavor, texture, and nutritional characteristics. Instead, the storage process of *E. macrophylla* resembles a stress-induced transformation process associated with dehydration and secondary metabolite redistribution.

Overall, the coordinated regulation of lipid, amino acid, and secondary metabolism demonstrates that *E. macrophylla* fruits undergo active metabolic remodeling rather than simple chemical degradation during storage. These metabolic characteristics highlight the distinct metabolic trajectory of *E. macrophylla* compared to typical fresh fruits and provide a basis for understanding how traditional storage processes may affect the chemical composition of medicinal–food plant resources.

Despite providing a comprehensive overview of metabolic changes during post-harvest storage, this study has several limitations. First, although significant correlations were observed between metabolite changes and antioxidant activity, causal relationships remain to be further established. The specific contributions of individual metabolites, particularly oxidized phenolic derivatives, to the changes in bioactivity require additional investigation. Second, the present study was conducted under a single storage condition (26 °C with shade-drying). Future studies should investigate the effects of different environmental factors, including temperature, humidity, and light exposure, on metabolic trajectories and product quality. Third, although this study characterized dynamic changes in metabolite composition during storage, the regulatory mechanisms that may govern these alterations at the transcriptional and post-translational levels remain to be elucidated. Integrating transcriptomics with metabolomics in future work could help to identify gene regulatory networks associated with these changes, potentially revealing candidate targets for targeted metabolic engineering or marker-assisted selection in breeding programs [58]. Notwithstanding these limitations, this research provides an indispensable foundation, transforming our understanding of *E. macrophylla* fruit from a static repository of compounds into a dynamic biochemical system whose therapeutic potential can be actively shaped through scientifically informed post-harvest science.

## 5. Conclusions

In this study, we demonstrate that the post-harvest storage of *E. macrophylla* fruit is characterized by phased metabolic changes rather than a simple degradation process. The 8-day storage point (T8) appears to be a pronounced metabolic transition, marking a shift in metabolite profiles from early stress-associated changes to an increased abundance of metabolites mapped to secondary metabolism pathways. Our metabolomic data are consistent with the hypothesis that the traditional color transition from yellow to black is associated with the oxidation, polymerization, and conversion of phenolic compounds, processes that may also contribute to changes in the fruit’s antioxidant profile. The core contribution of this work lies in its systematic delineation of the dynamic biochemical blueprint underlying a traditionally processed medicinal fruit. By moving beyond a static phytochemical inventory, our results reveal temporal changes in metabolites mapped to key biosynthetic routes, particularly those related to lipid diversification, alkaloid/terpenoid synthesis, and aromatic amino acid metabolism, suggesting coordinated metabolic shifts during storage. These findings contribute to the understanding of how post-harvest metabolic changes may influence phytochemical outcomes in traditionally processed fruits. This mechanistic insight transforms *E. macrophylla* from an empirical folk remedy into a model system for understanding how post-harvest biochemistry can be harnessed to direct phytochemical outcomes. Our research provides a scientific reference for informing post-harvest handling strategies aimed at preserving bioactive compounds and supporting the development of underutilized functional fruits for potential applications in the nutraceutical and pharmaceutical industries.

## Data Availability

The original contributions presented in this study are included in the article/Supplementary Materials. Further inquiries can be directed to the corresponding authors.

## Supplementary Materials

The following supporting information can be downloaded at: https://www.mdpi.com/article/10.3390/foods15173154/s1, Figure S1. Validation of differentially accumulated metabolites (DAMs) by targeted quantification; Figure S2. KEGG pathway enrichment analysis of down-regulated differentially accumulated metabolites (DAMs) in *E. macrophylla* fruits during postharvest storage. Table S1. Moisture content and cumulative weight loss of *E. macrophylla* fruits during storage at different time points; Table S2. Estimated LC-MS Peak Intensity of all Metabolites; Table S3. Analysis of differentially accumulated metabolites (DAMS) between the T4 vs T0 groups; Table S4. Analysis of differentially accumulated metabolites (DAMS) between the T8 vs T0 groups; Table S5. Analysis of differentially accumulated metabolites (DAMS) between the T12 vs T0 groups; Table S6. Analysis of differentially accumulated metabolites (DAMS) between the T8 vs T4 groups; Table S7. Analysis of differentially accumulated metabolites (DAMS) between the T12 vs T4 groups; Table S8. Analysis of differentially accumulated metabolites (DAMS) between the T12 vs T8 groups; Table S9. Summary of co-accumulation metabolite clusters.
