# Supplementary material for: Application of UPLC-MS/MS-Based Widely Targeted Metabolomics Reveals Metabolic Reprogramming During Post-Harvest Storage of Ehretia macrophylla Fruits
Source: Foods. 2026 Sep 5;15(17):3154. doi: 10.3390/foods15173154 (PMC13565585; doi:10.3390/foods15173154)
Supplement: Supplementary file 1 [file foods-15-03154-s001.zip › Figure S1.pdf]

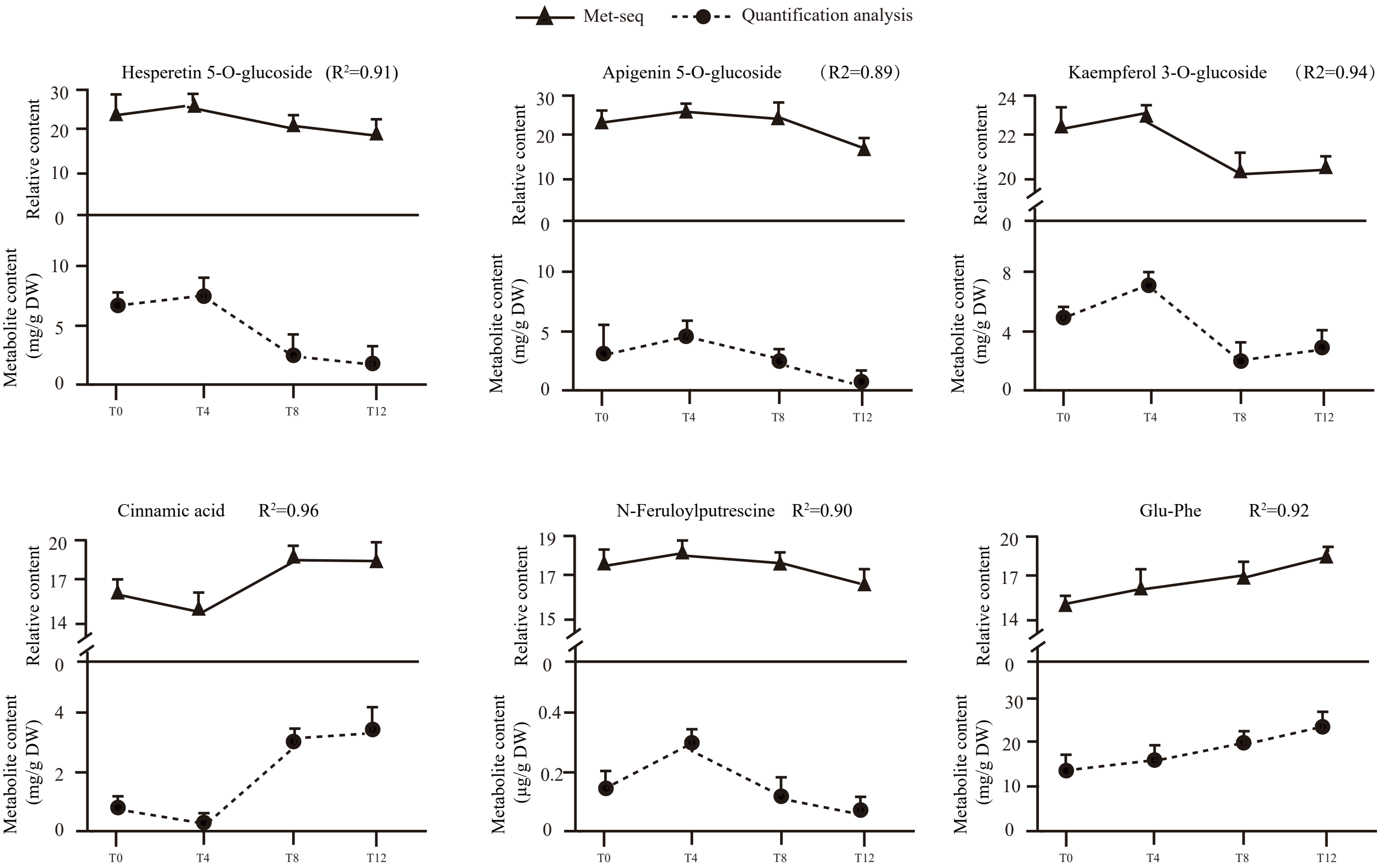

Met-seq
Quantification analysis
Hesperetin 5-O-glucoside (R²=0.91)
Apigenin 5-O-glucoside (R2=0.89)
Kaempferol 3-O-glucoside (R2=0.94)
Cinnamic acid R²=0.96
N-Feruloylputrescine R²=0.90
Glu-Phe R²=0.92
Relative content
Metabolite content (mg/g DW)
Metabolite content (μg/g DW)
T0
T4
T8
T12
