# Supplementary material for: Application of UPLC-MS/MS-Based Widely Targeted Metabolomics Reveals Metabolic Reprogramming During Post-Harvest Storage of Ehretia macrophylla Fruits
Source: Foods. 2026 Sep 5;15(17):3154. doi: 10.3390/foods15173154 (PMC13565585; doi:10.3390/foods15173154)
Supplement: Supplementary file 1 [file foods-15-03154-s001.zip › Figure S2.pdf]

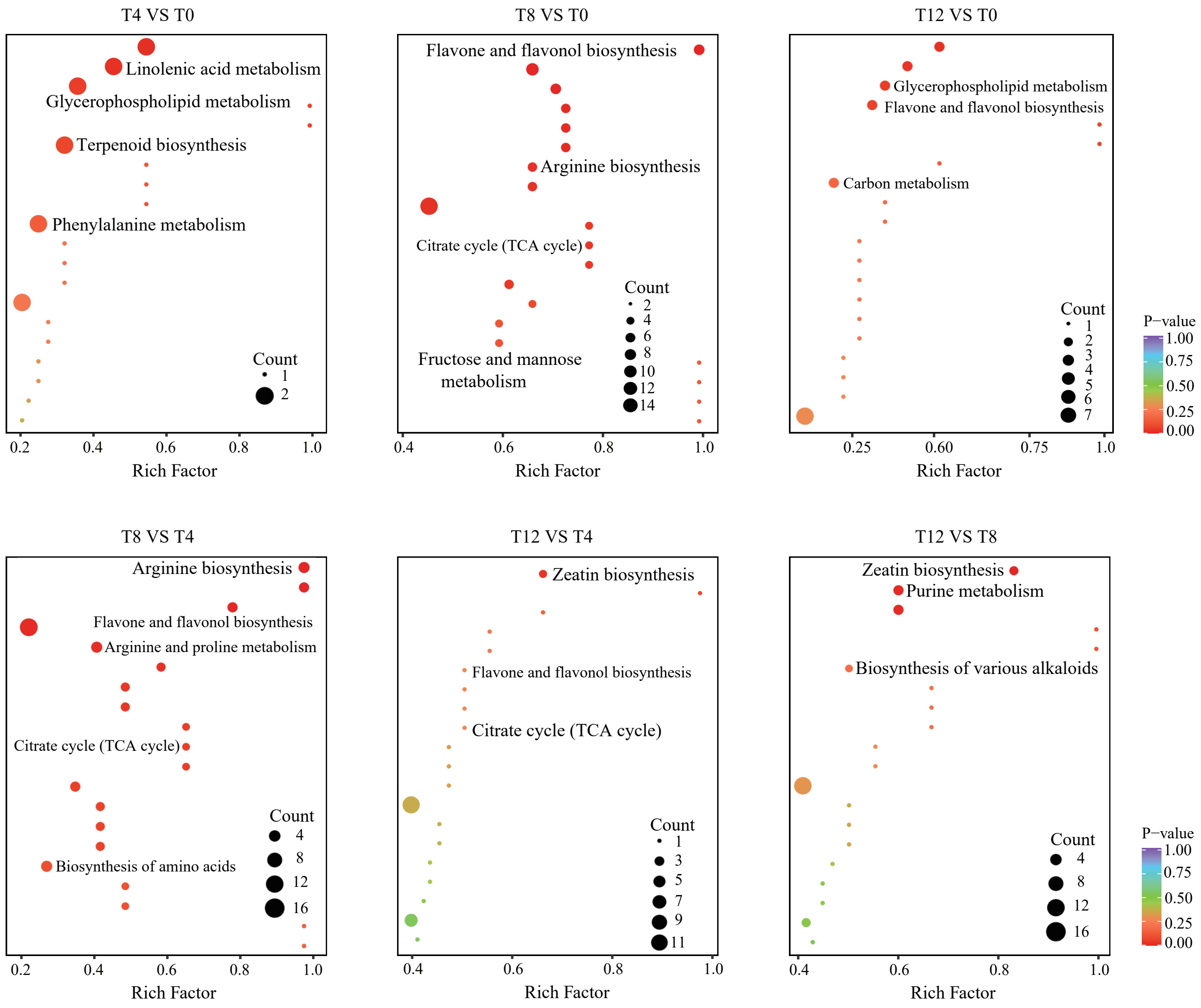
T4 VS T0
Linolenic acid metabolism
Glycerophospholipid metabolism
Terpenoid biosynthesis
Phenylalanine metabolism
Count
1
2
0.2
0.4
0.6
0.8
1.0
Rich Factor
T8 VS T0
Flavone and flavonol biosynthesis
Arginine biosynthesis
Citrate cycle (TCA cycle)
Fructose and mannose metabolism
Count
2
4
6
8
10
12
14
0.4
0.6
0.8
1.0
Rich Factor
T12 VS T0
Glycerophospholipid metabolism
Flavone and flavonol biosynthesis
Carbon metabolism
Count
1
2
3
4
5
6
7
0.25
0.60
0.75
1.0
Rich Factor
P−value
1.00
0.75
0.50
0.25
0.00
T8 VS T4
Arginine biosynthesis
Flavone and flavonol biosynthesis
Arginine and proline metabolism
Citrate cycle (TCA cycle)
Biosynthesis of amino acids
Count
4
8
12
16
0.2
0.4
0.6
0.8
1.0
Rich Factor
T12 VS T4
Zeatin biosynthesis
Flavone and flavonol biosynthesis
Citrate cycle (TCA cycle)
Count
1
3
5
7
9
11
0.4
0.6
0.8
1.0
Rich Factor
T12 VS T8
Zeatin biosynthesis
Purine metabolism
Biosynthesis of various alkaloids
Count
4
8
12
16
0.4
0.6
0.8
1.0
Rich Factor
P−value
1.00
0.75
0.50
0.25
0.00
