# Supplementary material for: Application of UPLC-MS/MS-Based Widely Targeted Metabolomics Reveals Metabolic Reprogramming During Post-Harvest Storage of Ehretia macrophylla Fruits
Source: Foods. 2026 Sep 5;15(17):3154. doi: 10.3390/foods15173154 (PMC13565585; doi:10.3390/foods15173154)
Supplement: Supplementary file 1 [file foods-15-03154-s001.zip › Supplementary.pdf]

Application of UPLC-MS/MS Based Widely Targeted Metabolomics Reveals Metabolic Reprogramming During Postharvest Storage of *Ehretia macrophylla* Fruits

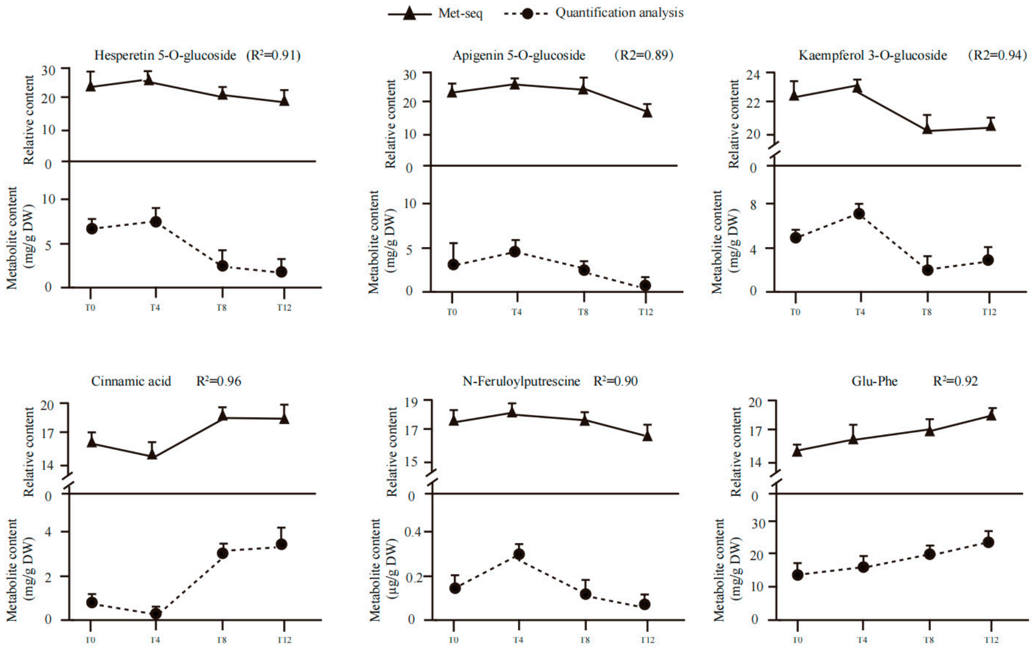

Figure S1. Validation of differentially accumulated metabolites (DAMs) by targeted quantification. The levels of selected DAMs identified in comparisons of T4, T8, and T12 versus T0 from the metabolomic data were quantified using external standards. Data are presented as mean ± standard deviation (n = 3 biological replicates). The consistency between relative abundance from metabolomics and absolute concentration from quantification was assessed by Pearson's correlation analysis, which revealed a strong and significant positive correlation ($r > 0.8$, $P < 0.05$).

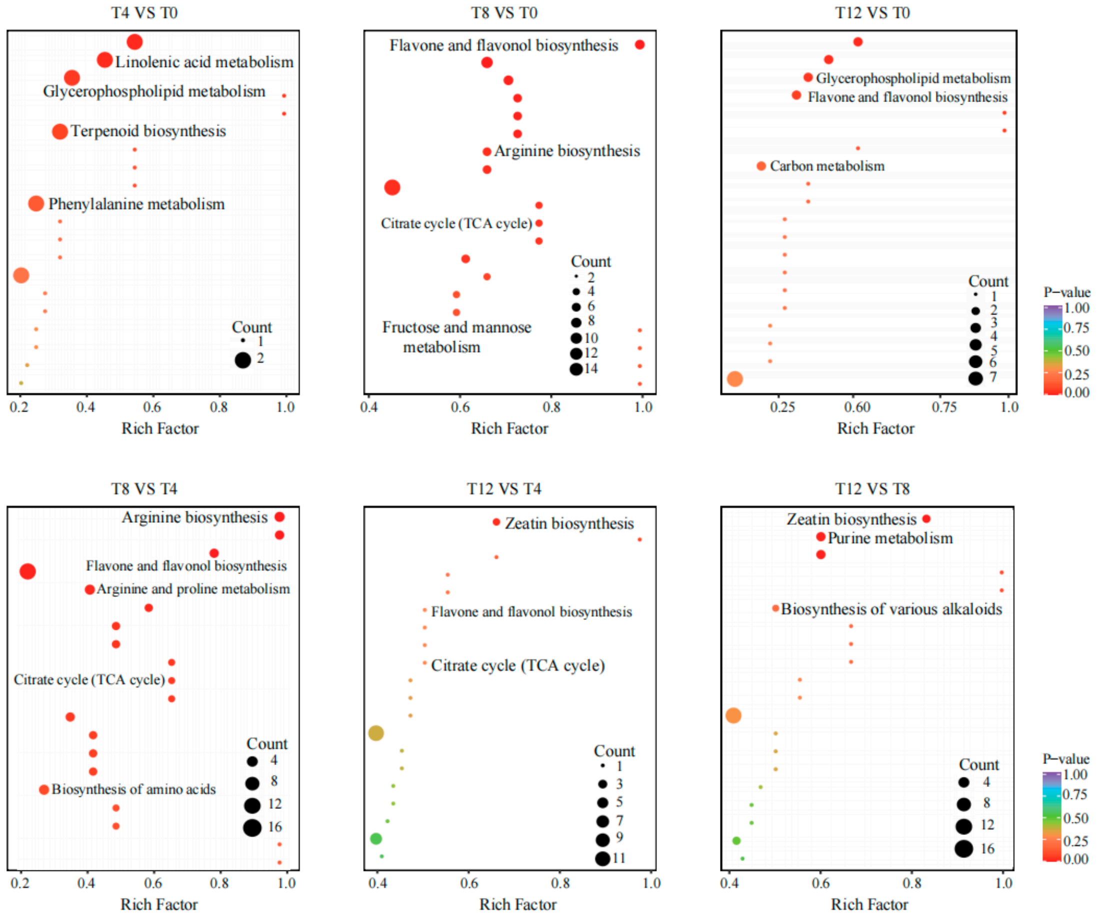

Figure S2. KEGG pathway enrichment analysis of down-regulated differentially accumulated metabolites (DAMs) in *E. macrophylla* fruits during postharvest storage. Bubble plots display significantly enriched metabolic pathways identified from pairwise comparisons across all four storage time points (six comparisons in total). For each comparison, only DAMs that were significantly down-regulated in the later time point relative to the earlier time point were subjected to enrichment analysis. The y-axis indicates KEGG pathway names, and the x-axis represents the enrichment factor. Bubble size corresponds to the number of down-regulated DAMs mapped to the respective pathway. Color intensity reflects the statistical significance of enrichment, with a gradient from blue to red.
